# The humanized platelet glycoprotein VI Fab inhibitor EMA601 protects from arterial thrombosis and ischaemic stroke in mice

**DOI:** 10.1093/eurheartj/ehae482

**Published:** 2024-08-16

**Authors:** Stefano Navarro, Ivan Talucci, Vanessa Göb, Stefanie Hartmann, Sarah Beck, Valerie Orth, Guido Stoll, Hans M Maric, David Stegner, Bernhard Nieswandt

**Affiliations:** Institute of Experimental Biomedicine I, Josef-Schneider-Straße 2, 97080 Würzburg, Germany; Rudolf Virchow Center, Center for Integrative and Translational Bioimaging, University of Wuerzburg, Josef-Schneider-Str. 2, 97080 Würzburg, Germany; Rudolf Virchow Center, Center for Integrative and Translational Bioimaging, University of Wuerzburg, Josef-Schneider-Str. 2, 97080 Würzburg, Germany; Department of Neurology, University Hospital Würzburg, Josef-Schneider-Str. 11, 97080 Würzburg, Germany; Institute of Experimental Biomedicine I, Josef-Schneider-Straße 2, 97080 Würzburg, Germany; Rudolf Virchow Center, Center for Integrative and Translational Bioimaging, University of Wuerzburg, Josef-Schneider-Str. 2, 97080 Würzburg, Germany; Institute of Experimental Biomedicine I, Josef-Schneider-Straße 2, 97080 Würzburg, Germany; Institute of Experimental Biomedicine I, Josef-Schneider-Straße 2, 97080 Würzburg, Germany; Rudolf Virchow Center, Center for Integrative and Translational Bioimaging, University of Wuerzburg, Josef-Schneider-Str. 2, 97080 Würzburg, Germany; EMFRET Analytics GmbH, Eibelstadt, Germany; Institute of Experimental Biomedicine I, Josef-Schneider-Straße 2, 97080 Würzburg, Germany; Rudolf Virchow Center, Center for Integrative and Translational Bioimaging, University of Wuerzburg, Josef-Schneider-Str. 2, 97080 Würzburg, Germany; Institute of Experimental Biomedicine I, Josef-Schneider-Straße 2, 97080 Würzburg, Germany; Rudolf Virchow Center, Center for Integrative and Translational Bioimaging, University of Wuerzburg, Josef-Schneider-Str. 2, 97080 Würzburg, Germany; Institute of Experimental Biomedicine I, Josef-Schneider-Straße 2, 97080 Würzburg, Germany; Rudolf Virchow Center, Center for Integrative and Translational Bioimaging, University of Wuerzburg, Josef-Schneider-Str. 2, 97080 Würzburg, Germany; EMFRET Analytics GmbH, Eibelstadt, Germany

**Keywords:** Platelet, GPVI, Anti-platelet therapy, Stroke, Thrombosis

## Abstract

**Background and Aims:**

Glycoprotein VI (GPVI) is a platelet collagen/fibrin(ogen) receptor and an emerging pharmacological target for the treatment of thrombotic and thrombo-inflammatory diseases, notably ischaemic stroke. A first anti-human GPVI (hGPVI) antibody Fab-fragment (ACT017/glenzocimab, *K*_D_: 4.1 nM) recently passed a clinical phase 1b/2a study in patients with acute ischaemic stroke and was found to be well tolerated, safe, and potentially beneficial. In this study, a novel humanized anti-GPVI antibody Fab-fragment (EMA601; *K*_D_: 0.195 nM) was developed that inhibits hGPVI function with very high potency *in vitro* and *in vivo*.

**Methods:**

Fab-fragments of the mouse anti-hGPVI IgG Emf6.1 were tested for functional GPVI inhibition in human platelets and in hGPVI expressing (*hGP6^tg/tg^*) mouse platelets. The *in vivo* effect of Emf6.1^Fab^ was assessed in a tail bleeding assay, an arterial thrombosis model and the transient middle cerebral artery occlusion (tMCAO) model of ischaemic stroke. Using complementary-determining region grafting, a humanized version of Emf6.1^Fab^ (EMA601) was generated. Emf6.1^Fab^/EMA601 interaction with hGPVI was mapped in array format and kinetics and quantified by bio-layer interferometry.

**Results:**

Emf6.1^Fab^ (*K*_D_: 0.427 nM) blocked GPVI function in human and *hGP6^tg/tg^* mouse platelets in multiple assays *in vitro* at concentrations ≥5 µg/mL. Emf6.1^Fab^ (4 mg/kg)-treated *hGP6^tg/tg^* mice showed potent hGPVI inhibition *ex vivo* and were profoundly protected from arterial thrombosis as well as from cerebral infarct growth after tMCAO, whereas tail-bleeding times remained unaffected. Emf6.1^Fab^ binds to a so far undescribed membrane proximal epitope in GPVI. The humanized variant EMA601 displayed further increased affinity for hGPVI (*K*_D_: 0.195 nM) and fully inhibited the receptor at 0.5 µg/mL, corresponding to a >50-fold potency compared with ACT017.

**Conclusions:**

EMA601 is a conceptually novel and promising anti-platelet agent to efficiently prevent or treat arterial thrombosis and thrombo-inflammatory pathologies in humans at risk.

## Structured Graphical Abstract

**Structured Graphical Abstract:**
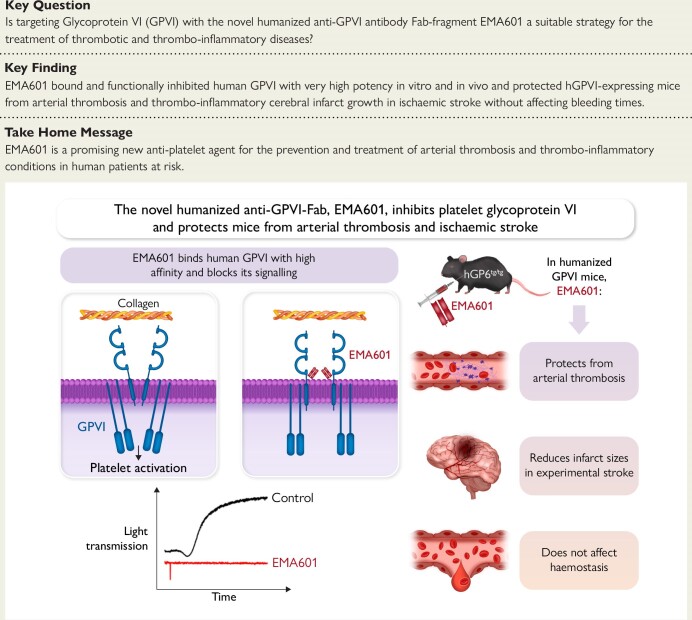
The novel humanized anti-glycoprotein VI (GPVI)-Fab, EMA601, very potently inhibits human GPVI signalling without blocking the ligand-binding site of the receptor. *In vivo*, EMA601 protects from arterial thrombosis and ischaemic stroke, while leaving tail bleeding times unaffected.

**See the editorial comment for this article ‘The quest for the holy grail in antithrombotic therapy: revitalized hope for platelet GPVI as a safe and effective antithrombotic target’, by J.D. McFadyen *et al.*, https://doi.org/10.1093/eurheartj/ehae592.**

## Introduction

Platelet adhesion and aggregation at sites of vascular injury is essential for normal haemostasis and maintenance of vascular integrity.^1^ The pathological deviation of haemostasis is thrombosis where intravascular clots form in an uncontrolled manner, e.g. at sites of atherosclerotic plaque rupture. This can result in vascular occlusion and cause life-threatening disease states, such as myocardial infarction or acute ischaemic stroke (AIS), which represent the leading causes of death and severe disability and remain a major global health burden.^2^ Therefore, anti-platelet drugs, such as acetyl salicylic acid, P2Y_12_ adenosine diphosphate (ADP) receptor blockers, or glycoprotein (GP) IIb/IIIa inhibitors, have become indispensable therapeutics to efficiently prevent or treat arterial thrombosis, but they all carry an inherent risk of bleeding, most obvious in multimorbid patients requiring dual platelet inhibition or combined anti-coagulation.^2–4^ The development of new anti-thrombotic treatment regimens with a focus on a reduced risk of bleeding without losing drug efficacy, however, remains challenging.

Among platelet receptors, GPVI is considered a promising pharmacological target, as both its absence and its functional inhibition provide protection from arterial thrombosis and thrombo-inflammatory pathologies, such as (hyper-)acute ischaemic stroke^5–9^ or lipopolysaccharide (LPS)-induced acute lung injury^10^ in several mammalian model organisms without causing major bleeding complications.^6,11–13^

Glycoprotein VI is a ∼65 kDa receptor for collagen and fibrin that is exclusively expressed on platelets and megakaryocytes. It is non-covalently associated with the signal transducing immunoreceptor tyrosine-based activation motif-containing Fc receptor γ-subunit (FcRγ-chain).^14^ The GPVI ectodomain comprises two immunoglobulin (Ig)-like domains, namely D1 and D2, followed by an assumably intrinsically disordered region proximal to the trans-membrane region. X-ray crystallography attributed collagen binding to D1,^15^ whereas GPVI dimerization has been proposed to be mediated by D2 and parts of the following disordered region.^16^

Different approaches to pharmacologically target GPVI-mediated platelet activation have been reported, including antibody (IgG)-mediated GPVI immunodepletion,^11,17^ competitive inhibition by a dimeric GPVI-Fc fusion protein (Revacept^18,19^), and GPVI blockade by monovalent IgG-derived Fab fragments,^17,20^ and all studies showed a significant benefit of these different anti-GPVI strategies in models of thrombosis and thrombo-inflammation.

Based on these promising results, two conceptually different inhibitors of GPVI function have been developed and tested in clinical Phase 1/2 trials. While the GPVI competitor Revacept was found to be safe, it failed to reduce the incidence of myocardial injury in patients with stable ischaemic heart disease,^19^ which may be explained by its lower GPVI inhibiting effect compared with agents directly targeting the receptor.^21,22^ Indeed, rather promising results were recently reported from a clinical Phase 1b/2a study in patients with AIS, showing that the humanized GPVI-inhibitory Fab ACT017 (glenzocimab), when given in addition to thrombolysis/endovascular thrombectomy (EVT), was well tolerated and may reduce serious adverse events, intracranial haemorrhage, and mortality.^23^ These data indicated that GPVI inhibitors might attenuate the thrombo-inflammatory response that drives cerebral infarct progression in (hyper-) acute stroke, as previously shown in experimental stroke models.^5–9^

Initial studies with the parent mouse IgG-Fab of ACT017, 9.O12^Fab^,^24^ had shown significant GPVI inhibition *ex vivo* in non-human primates^25^ and in human GPVI (hGPVI) expressing mice.^26^ However, the *in vivo* inhibitory effect of 9.O12^Fab^ was comparably short-lived in both species which could, at least partly, be explained by the rather moderate binding affinity of the antibody to GPVI (*K*_D_: 17.08 nM).^24,25,27^ Mapping of the 9.O12/ACT017 binding site on GPVI revealed a discontinuous epitope composed of two stretches 114–142 and 165–187 of hGPVI (numbering according to Q9HCN6) located in the D2 domain.^28^ Up to date, >30 antibodies and derivates thereof have been reported, and their GPVI binding sites have been mapped on regions between residues 58 and 187, but not beyond.^28^

Here, we report the development of novel anti-hGPVI antibody Fab fragment (Emf6.1^Fab^) and its fully humanized variant, EMA601 that bind to a so-far undescribed membrane-proximal epitope in the receptor and block its function with very high potency. Emf6.1^Fab^ treatment of *hGP6^tg/tg^* mice^29^ resulted in sustained GPVI inhibition and marked protection from arterial thrombosis and ischaemic stroke while not affecting tail bleeding times.

## Methods

Detailed methodology is provided in Supplementary data online, *Methods*.

### Animals

Animal experiments were approved by the district government of Lower Franconia (Regierung von Unterfranken) and performed in accordance with the current Animal Research: Reporting of in Vivo Experiments guidelines (https://arriveguidelines.org/). Mice were matched for age, sex, and genetic background. The mouse line humanized for *GP6* (*hGP6^tg/tg^*) has been reported previously.^29^

### Transient middle cerebral artery occlusion and infarct size measurment

Ten- to 14-week-old, male *hGP6^tg/tg^* mice were injected i.v. with 4 mg/kg Emf6.1^Fab^ 1 h before surgery. Mice were subjected to the transient middle cerebral artery occlusion (tMCAO) model and received a second dose of 4 mg/kg Emf6.1^Fab^ s.c. after 6 h. At 24 h after tMCAO, infarct sizes were determined, as previously described.^30,31^

### Arterial thrombosis models

*Mechanical injury of the abdominal aorta*: The aorta of anaesthetized mice (10–12 weeks old) was exposed, a Doppler ultrasonic flow probe was placed around the vessel. Injury was induced by compressing the aorta for 5 s with clamps. Blood flow was monitored over 30 min or until complete occlusion occurred (blood flow stopped for >5 min).

*FeCl_3_-induced carotid artery thrombosis*: The left carotid artery of anaesthetized mice (10–12 weeks old) was exposed, and the flow probe was placed around the vessel. Thrombus formation was induced by a filter paper saturated with 10% FeCl_3_ and placed on the carotid artery for 3 min. Blood flow was monitored over 30 min or until complete occlusion occurred (blood flow stopped for >5 min).

### Statistics

Statistical analyses were conducted using GraphPad Prism version 9.0.0 (GraphPad Software). Data distribution was analysed using the Shapiro–Wilk test. Depending on the distribution differences were evaluated using the Mann–Whitney *U* test, Kruskal–Wallis test for one-way analysis, or a two-way analysis of variance with Bonferroni *post hoc* test. Fisher’s exact test was employed for categorical data. Statistical significance was set at *P* ≤ .05. Data are expressed as mean ± standard deviation.

## Results

### Emf6.1 binds to human glycoprotein VI and blocks its function

A series of monoclonal mouse IgG antibodies against hGPVI (Emf antibodies) was generated by standard hybridoma technology and 16 clones were isolated based on their specific binding to the hGPVI ectodomain (Emf1–Emf16). Flow cytometric analysis showed that nine of them also bound to human platelets, i.e. membrane-expressed GPVI, among them Emf1, Emf2, Emf3, and Emf6.1 (Supplementary data online, *Figure S1A*). These clones were further characterized for their ability to inhibit GPVI function. Emf1,^20^ Emf3, and Emf6.1 efficiently inhibited collagen-related peptide (CRP)- and collagen-induced responses of human platelets with Emf6.1 (mouse IgG2a κ), consistently showing the most potent inhibitory effect. Emf6.1-IgG (10 µg/mL) abolished CRP- and collagen-induced aggregate formation, whereas it had no effect on responses to other agonists (Supplementary data online, *Figure S1B*), and it potently blocked aggregate formation of human platelets on collagen under flow (1000 s^−1^) in a whole-blood perfusion assay when added at 10, 5, 2, or 1 µg/mL *in vitro* (Supplementary data online, *Figure S1C*–*E*). To circumvent possible Fc-mediated platelet activation by Emf6.1-IgG and to exclude Fc-dependent thrombocytopenia and/or GPVI depletion,^11^ Fab fragments of Emf6.1 were generated and used for all further studies.

### Emf6.1^Fab^ inhibits glycoprotein VI–dependent activation of human platelets

**Figure 1:**
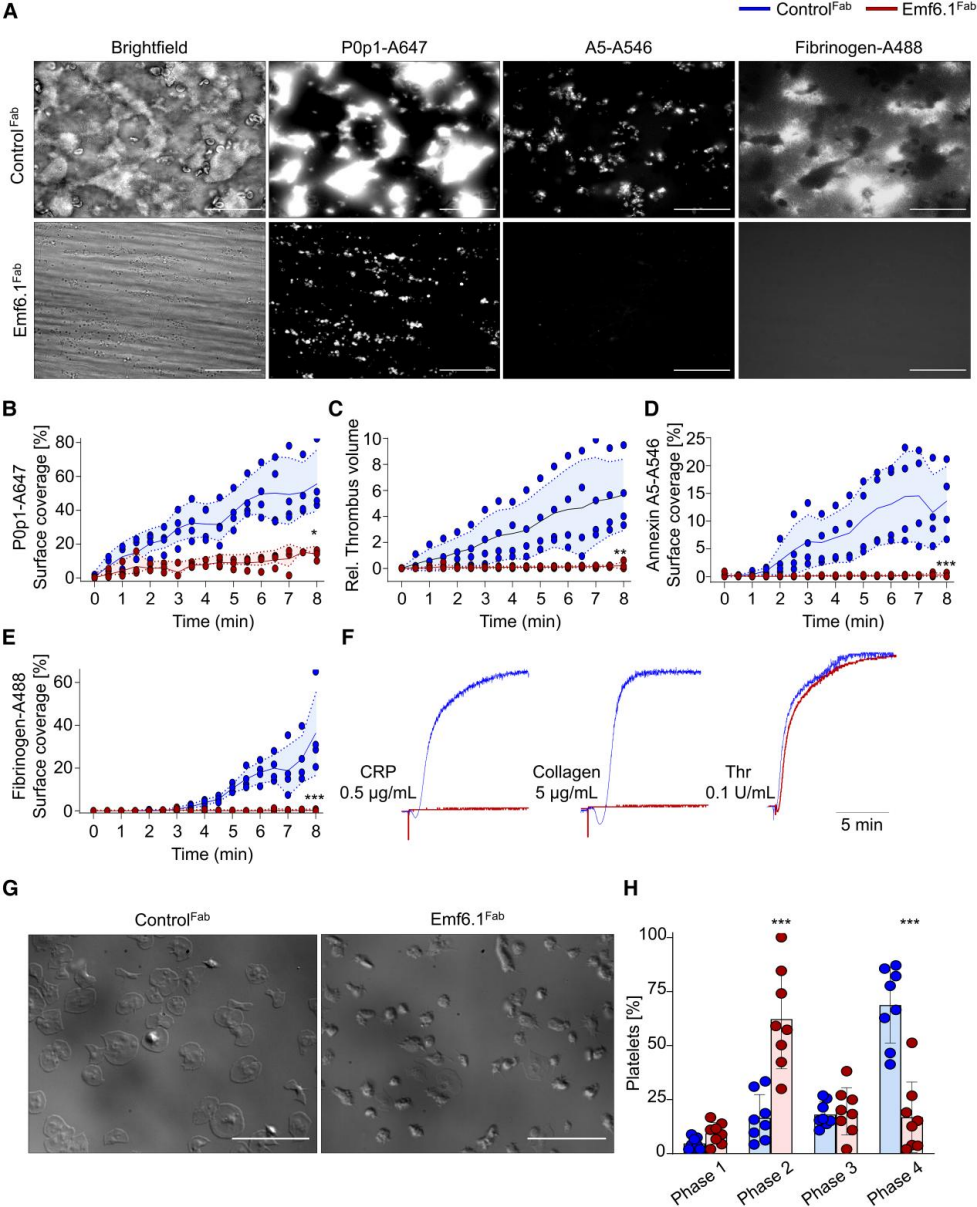
Emf6.1^Fab^ inhibits glycoprotein VI–induced aggregation, thrombus formation, and spreading of human platelets. (*A–E*) Citrated blood from healthy donors was incubated with 5 µg/mL control Fab or Emf6.1^Fab^ and then perfused over a collagen and tissue factor–coated surface (shear: 1000s^−1^). (*A*) Representative images; scale bar 50 µm. Platelet adhesion (*B*), thrombus formation (*C*), phosphatidylserine exposure (*D*), and fibrin/fibrinogen deposition (*E*) were assessed. Collagen and tissue factor were coated at 50 µg/mL and 500 pM, respectively. A flow rate of 1000 s^−1^ was used. Values are mean ± standard deviation (*n* = 4). A5: Annexin V, unpaired Mann–Whitney *U* test. **P < .01, ***P < .001. (*F*) Aggregation responses to the indicated agonists of washed human platelets pre-treated with 10 µg/mL Emf6.1^Fab^ or control Fab in light-transmission aggregometry (*n* = 4). (*G* and *H*) Washed human platelets were allowed to spread on fibrinogen (100 µg/mL) for 45 min at 37°C. DIC pictures were taken (100× objective; scale bar 30 µm) (*G*) and phase abundance was determined. Phase 1: adhesion; Phase 2: filopodia formation; Phase 3: lamellipodia formation; Phase 4: fully spread platelet (*H*). Values are mean ± standard deviation (*n* = 8); unpaired, Mann–Whitney *U* test. ***P < .001

Emf6.1^Fab^ (10 µg/mL) almost completely abrogated human platelet adhesion and aggregate formation on collagen at a shear rate of 1000 s^−1^ (Supplementary data online, *Figure S2A–C*). Notably, also at lower concentrations of Emf6.1^Fab^ (5, 2, and 1 µg/mL), aggregate formation was potently inhibited, with a ∼75% reduction in thrombus volume compared with control^Fab^ at 1 µg/mL (Supplementary data online, *Figure S2A*–*I*). We also evaluated the effect of Emf6.1^Fab^ on thrombus formation of human platelets on collagen/tissue factor (TF) spots in recalcified whole blood under flow.^32^ Under these conditions, 5 µg/mL Emf6.1^Fab^ potently inhibited platelet deposition and thrombus formation (*Figure 1A–C*) and abolished phosphatidylserine (PS) exposure and fibrin/fibrinogen deposition (*Figure 1A, D, and E*).

Furthermore, Emf6.1^Fab^ (5 µg/mL) blocked CRP- and collagen-induced aggregation, while thrombin-induced responses remained unaltered (*Figure 1F*). Dose–response experiments showed that even at a concentration of 1 µg/mL Emf6.1^Fab^ completely blocked CRP-induced aggregation and markedly reduced collagen-induced aggregation (Supplementary data online, *Figure S2J*). Human GPVI facilitates platelet activation and spreading on immobilized fibrinogen.^33^ This response was also potently inhibited by Emf6.1^Fab^ (10 µg/mL; *Figure 1G and H*). Together, these data showed that Emf6.1^Fab^ potently inhibits GPVI-dependent activation, aggregation, and spreading of human platelets *in vitro*.

### Emf6.1^Fab^ fully inhibits glycoprotein VI function in *hGP6^tg/tg^* mouse platelets

To study the effect of Emf6.1^Fab^*in vivo*, we capitalized on a mouse line humanized for GPVI (*hGP6^tg/tg^*).^29^ Flow cytometric analysis showed that Emf6.1^Fab^ (5 µg/mL) abolished CRP-induced activation, but had no effect on the responses to other agonists, such as thrombin, ADP, or the thromboxane A2 analogue U46619 (Supplementary data online, *Figure S3A and B*).

**Figure 2:**
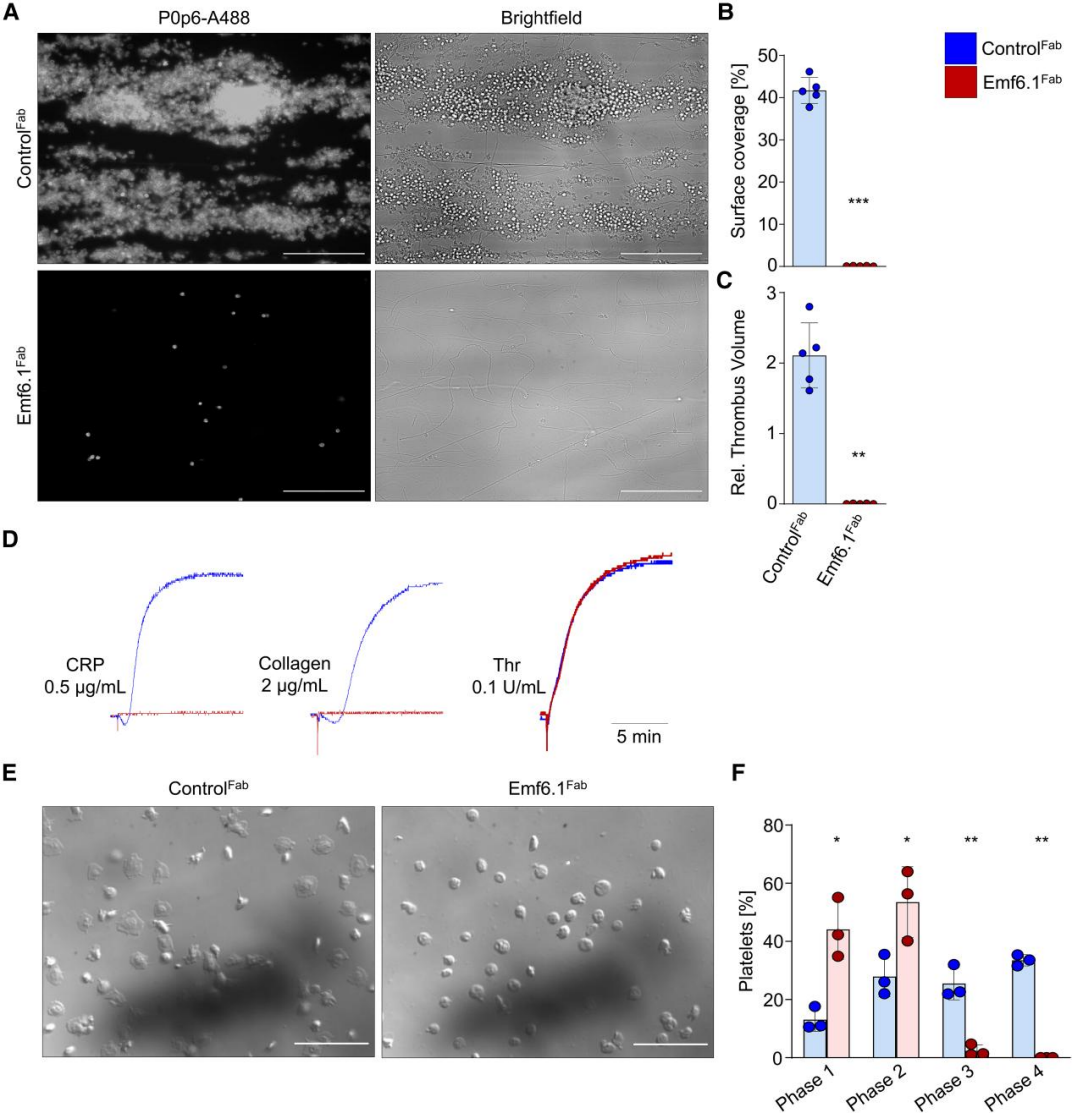
Emf6.1^Fab^ inhibits glycoprotein VI–mediated aggregation, thrombus formation, and spreading of *hGP6^tg/tg^* platelets. (*A–C*) Assessment of platelet adhesion (*B*) and aggregate formation (*C*) on Horm collagen (200 µg/mL) under flow (1000 s^−1^) in heparinized *hGP6^tg/tg^* blood treated with 2 µg/mL of either Emf6.1^Fab^ or control Fab. (*A*) Representative images are shown, scale 50 µm. Unpaired Mann–Whitney *U* test; values are mean ± standard deviation (*n* = 5); **P < .01, ***P < .001. (*D*) Aggregation responses of washed *hGP6^tg/tg^* platelets treated with either 5 µg/mL Emf6.1^Fab^ or control Fab in light-transmission aggregometry (*n* = 5). Thr: Thrombin. (*E* and *F*) Washed *hGP6^tg/tg^* platelets were allowed to spread on fibrinogen (100 µg/mL) for 45 min at 37°C. DIC pictures were taken (100× objective, scale 30 µm) (*E*) and phase abundance was determined. Phase 1: adhesion; Phase 2: filopodia formation; Phase 3: lamellipodia formation; Phase 4: fully spread platelet (*F*). Values are mean ± standard deviation (*n* = 3); unpaired, Mann–Whitney *U* test.*P < .05, **P < .01

Similar to human platelets, 2 µg/mL Emf6.1^Fab^ fully inhibited aggregate formation of *hGP6^tg/tg^* platelets on collagen in whole-blood perfusion studies (1000 s^−1^; *Figure 2A–C*). Furthermore, Emf6.1^Fab^ (5 or 2 µg/mL) almost completely inhibited GPVI-dependent aggregation of *hGP6^tg/tg^* platelets (*Figure 2D* and Supplementary data online, *Figure S3C*). It has been shown that mouse platelets expressing hGPVI, but not wild-type (WT) platelets, can fully spread on fibrinogen.^29,33^ Indeed, *hGP6^tg/tg^* platelets successfully spread on the fibrinogen-coated surface, and this process was abolished in the presence of Emf6.1^Fab^ (*Figure 2E and F*). Together, these data showed comparable *in vitro* effects of Emf6.1^Fab^ on human and *hGP6^tg/tg^* mouse platelets, thus confirming previous results^29^ showing that the mouse line is suitable to study the *in vivo* effects of hGPVI inhibitors.

### Emf6.1^Fab^ efficiently blocks glycoprotein VI function in *hGP6^tg/tg^* mice *ex vivo*

**Figure 3:**
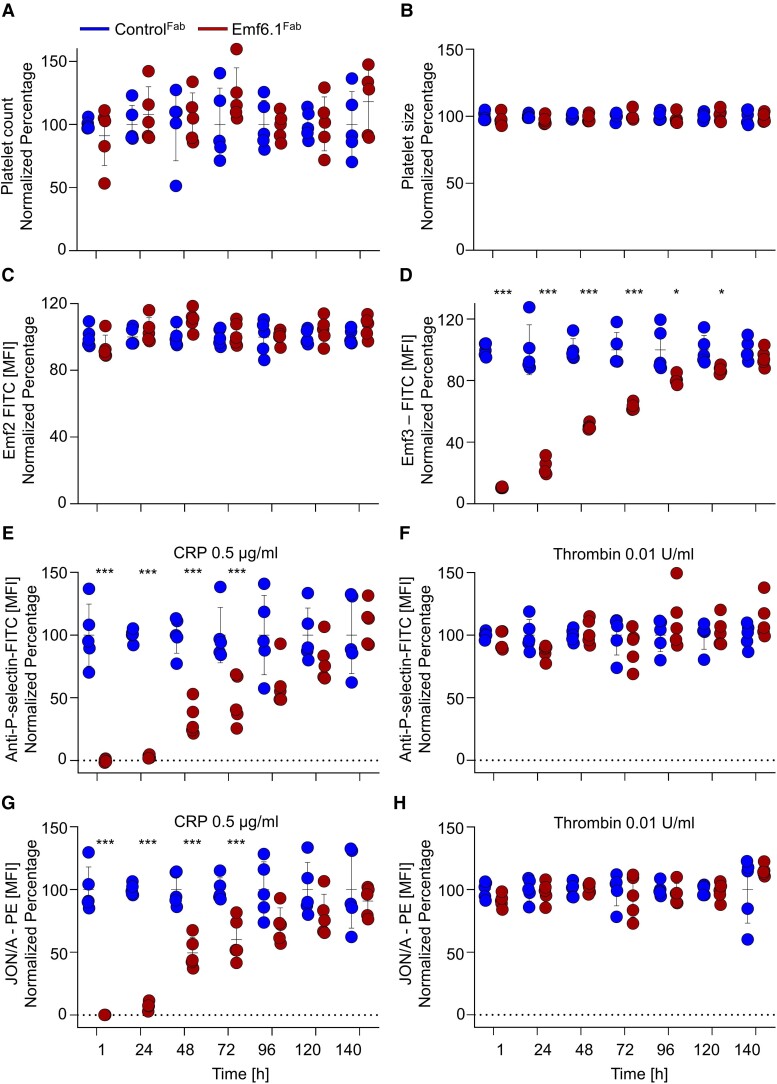
Emf6.1^Fab^ efficiently blocks glycoprotein VI function in *hGP6^tg/tg^* mice. *hGP6^tg/tg^* animals (*n* = 5) were treated with 4 mg/kg b.w. Emf6.1^Fab^ or control Fab. (*A* and *B*) Platelet count (*A*) and size (*B*) were determined using an automated cell counter. (*C* and *D*) Glycoprotein VI exposure was tested using Emf2 IgG-FITC (*C*), whereas epitope saturation of Emf6.1 was tested using Emf3 IgG-FITC (*D*). (*E–H*) Degranulation (α-P-selectin-FITC) (*E* and *F*) and activation of platelet αIIbβ3 integrin (JON/A-PE) (*G* and *H*) in 4 mg/kg b.w. Emf6.1^Fab^ or control Fab-treated *hGP6^tg/tg^* mice was determined in flow cytometry upon activation with the indicated agonists. Data are expressed as mean ± standard deviation; significance is expressed as *P < .05, ***P < .001, vs. indicated group (two-way analysis of variance followed by Bonferroni’s multiple comparison test; *n* = 5)

To study the *in vivo* effects of Emf6.1^Fab^, *hGP6^tg/tg^* mice received a dose of 4 mg/kg intravenously and their peripheral platelets were monitored for 5 days by flow cytometry and automated cell analysis. Platelet count and size were unaltered compared with control at all tested time points (*Figure 3A and B*), and no alteration of GPVI surface levels were detectable (*Figure 3C*). Next, we assessed GPVI occupancy by Emf6.1^Fab^ on circulating platelets over time using a fluorescein isothiocyanate (FITC)-labelled anti-GPVI mAb competing for the Emf6.1 binding site (Emf3). We detected significant epitope occupancy for up to 96 h after injection of Emf6.1^Fab^ (*Figure 3D*). In detail, after 1 h, ∼90% of the binding epitopes were occupied by Emf6.1^Fab^ and the percentage slowly declined to ∼76% at 24 h, ∼50% at 48 h, ∼36% at 72 h, ∼19% at 96 h, and ∼13% at 120 h. This sustained GPVI blockade was also functionally confirmed by measuring CRP-induced platelet activation in diluted blood *ex vivo*. The activation response was abolished at 1 and 24 h and still significantly reduced for up to 72 h in Emf6.1^Fab^ compared with control^Fab^-treated mice (*Figure 3E and G*). Thrombin-induced activation was indistinguishable between the two groups at all time points (*Figure 3F and H*).

**Figure 4:**
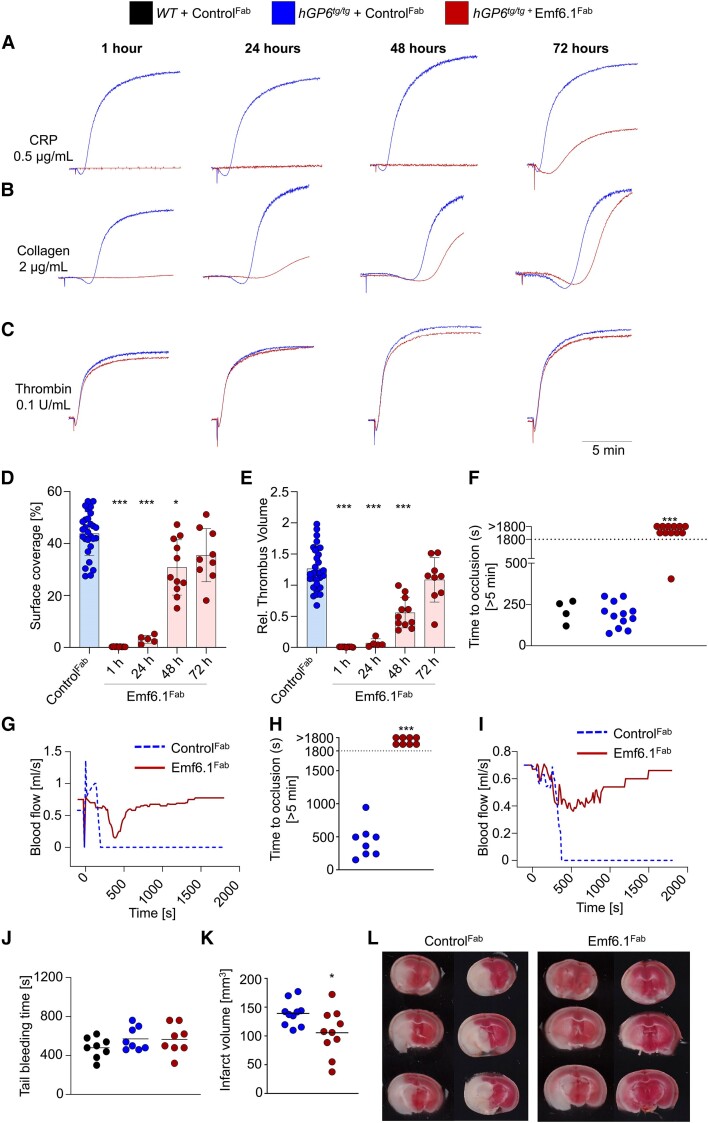
Emf6.1^Fab^ treatment (4 mg/kg b.w.) protects mice from occlusive arterial thrombosis and ischaemic stroke without impairing haemostasis. (*A–C*) Aggregation responses of washed platelets from *hGP6^tg/tg^* mice intravenously treated with 4 mg/kg b.w. Emf6.1^Fab^ or control Fab to collagen-related peptide (*A*), collagen (*B*), or thrombin (*C*) in light-transmission aggregometry (*n* = 6). (*D* and *E*) Assessment of platelet adhesion (*D*) and aggregate formation (*E*) on Horm collagen (200 µg/mL) under flow (1000 s^−1^) in heparinized blood from *hGP6^tg/tg^* mice intravenously treated with 4 mg/kg b.w. Emf6.1^Fab^ or control Fab solution at different time points after injection. Values are mean ± standard deviation. Two-way analysis of variance followed by Bonferroni’s multiple comparison test *P < .05, ***P < .001. (*F* and *G*). Arterial thrombosis was assessed for a maximum period of 30 min after control Fab or Emf6.1^Fab^ treatment in the mechanical injury of the aorta thrombosis model. (*F*) Time to vessel occlusion is depicted with each symbol representing one animal. ***P < .001 using Fisher’s exact test. (*G*) Representative blood flow traces are shown from control Fab and Emf6.1^Fab^-treated mice. (*H* and *I*) Arterial thrombosis was assessed for a maximum period of 30 min after control Fab or Emf6.1^Fab^ treatment by topically injuring the carotid artery with a saturated filter paper with 10% FeCl_3_ for 3 min and time to occlusion was determined (*H*). Each symbol represents one individual. Representative blood flow traces are shown from control Fab and Emf6.1^Fab^-treated mice (*I*). ***P < .001 using Fisher’s exact test. (*J*) Haemostasis was assessed using the tail bleeding time with each symbol representing one mouse. (*K* and *L*) Cerebral infarct development after experimental stroke. (*K*) Oedema-corrected quantifications of infarct sizes from mice either treated with 4 mg/kg Emf6.1^Fab^ or control before transient middle cerebral artery occlusion. (*L*) Representative images of brain sections stained with triphenyltetrazolium chloride to visualize infarcts. Each column of images represents three consecutive sections of one mouse. Data are depicted as mean ± standard deviation. Significance is expressed as *P < .05 using the Mann–Whitney *U* test

We also found a marked inhibition of CRP-induced platelet aggregation *ex vivo* for up to 72 h after injection of 4 mg/kg Emf6.1^Fab^, but not control^Fab^ (*Figure 4A*). Also, collagen-induced aggregation was potently blocked by the Emf6.1^Fab^ treatment, showing complete inhibition at 1 h and >80% at 24 h, which progressively declined at 48 and 72 h (*Figure 4B*). As expected, thrombin-induced aggregation was unaltered at all tested time points (*Figure 4C*). The robust inhibitory effect of Emf6.1^Fab^*ex vivo* was also seen in the whole-blood perfusion assay (1000 s^−1^) on collagen, showing a virtually complete inhibition of thrombus formation at 1 and 24 h after Emf6.1^Fab^ treatment. Furthermore, a significant reduction of surface coverage and thrombus volume was still detectable at 48 h after treatment (*Figure 4D and E* and Supplementary data online, *Figure S4*). Together, these data showed Emf6.1^Fab^ treatment of *hGP6^tg/tg^* mice resulted in profound and sustained inhibition of GPVI-dependent platelet activation *ex vivo*.

### Emf6.1^Fab^ protects *hGP6^tg/tg^* mice from arterial thrombosis and neurological damage in ischaemic stroke

The antithrombotic potential of Emf6.1^Fab^ was tested in two models of arterial thrombosis. In the first model, the abdominal aorta is mechanically injured and blood flow/occlusive thrombus formation is monitored by an ultrasonic flow probe.^34,35^ In this setting, Emf6.1^Fab^-treated mice (1 h after injection of 4 mg/kg) were strongly protected from thrombotic vessel occlusion (*Figure 4F*) with 91.7% (11 of 12) not forming a stable thrombus within the observation period of 30 min, whereas 100% of the controls occluded. In most of the Emf6.1^Fab^-treated mice, a transient reduction of the blood flow was detectable, indicative of transient and instable thrombus formation (*Figure 4G*).

A similar protective effect of Emf6.1^Fab^ treatment was found in a model of FeCl_3_-induced carotid artery thrombosis. While all (8/8) arteries occluded in control^Fab^-treated mice, Emf6.1^Fab^-treated mice were protected (0/8 occluded; *Figure 4H and I*).

Next, we assessed the effect of Emf6.1^Fab^ and control^Fab^ treatment in a tail bleeding assay and found no difference between the two groups of *hGP6^tg/tg^* mice (*Figure 4J*). Since a GPVI inhibitor may be used in combination with other antithrombotic or fibrinolytic drugs, we also assessed tail bleeding times in mice treated with ASA (Lyso-acetylsalicate 1.5 mg/kg i.v.) or rt-PA (0.9 mg/kg i.v.) in combination with Emf6.1^Fab^ or control^Fab^ (4 mg/kg each). Also under these conditions, Emf6.1^Fab^ treatment did not induce a significant prolongation of bleeding time (Supplementary data online, *Figure S4B*), thus confirming the strong safety profile of the Fab fragment.

Previous work has shown that the genetic or antibody-induced ablation of GPVI expression leads to a mild bleeding defect in humans and moderately increased tail bleeding times in mice,^11,14,36,37^ respectively. In line with these previous findings, depletion of hGPVI in *hGP6^tg/tg^* mice by Emf1-IgG treatment resulted in a moderate but significant increase in tail bleeding time (Supplementary data online, *Figure S4C*–*F*). Thus, the absence of GPVI results in a haemostatic defect that is not seen upon GPVI inhibition by Emf6.1^Fab^, suggesting that the receptor at least partly retained its adhesive/haemostatic function in the presence of the Fab. To test this directly, we performed whole-blood perfusion assays on collagen with hGPVI-depleted (4 mg/kg Emf1-IgG 5 days before analysis) vs. GPVI-blocked (10 µg/mL Emf6.1^Fab^) *hGP6^tg/tg^* blood and analysed platelet adhesion to the substrate at *t* = 2 min, i.e. in the absence of a washing step. Under these experimental conditions, the number of adherent platelets was ∼10-fold higher in the Emf6.1^Fab^-treated samples compared with hGPVI-deficient samples, strongly indicating that hGPVI, at least partly, retained its adhesive function in the presence Emf6.1^Fab^ (Supplementary data online, *Figure S4G and H*).

Previous studies have shown that the absence of GPVI protects mice from thrombo-inflammatory cerebral infarct growth in models of experimental stroke.^5–8^ Remarkably, however, although ACT017 has recently been tested in a clinical Phase 1b/2a trial in patients with AIS,^23^ no data on a therapeutic effect of Fab-mediated GPVI inhibition in experimental stroke have been reported to date.

To test this directly, *hGP6^tg/tg^* mice received Emf6.1^Fab^ or control^Fab^ (4 mg/kg i.v.) and were subjected to 1 h of tMCAO followed by 23 h reperfusion. At *t* = 6 h after tMCAO, the animals received a second injection (4 mg/kg s.c.) of the respective Fab. Strikingly, infarct volumes in the Emf6.1^Fab^-treated mice were significantly reduced compared with control^Fab^-treated littermates at 24 h after tMCAO as measured by triphenyltetrazolium chloride (TTC) staining [Med.: 105.9 (25%: 88.5; 75%: 121.0) vs. 137.1 (25%: 119.6; 75%: 142.3) mm^3^; *P* < .05; *Figure 4K and L*]. Thus, the functional inhibition of hGPVI by Emf6.1^Fab^ efficiently attenuated thrombo-inflammatory cerebral infarct growth after tMCAO.

### Emf6.1 binds to the putative glycoprotein VI-dimerization site

**Figure 5:**
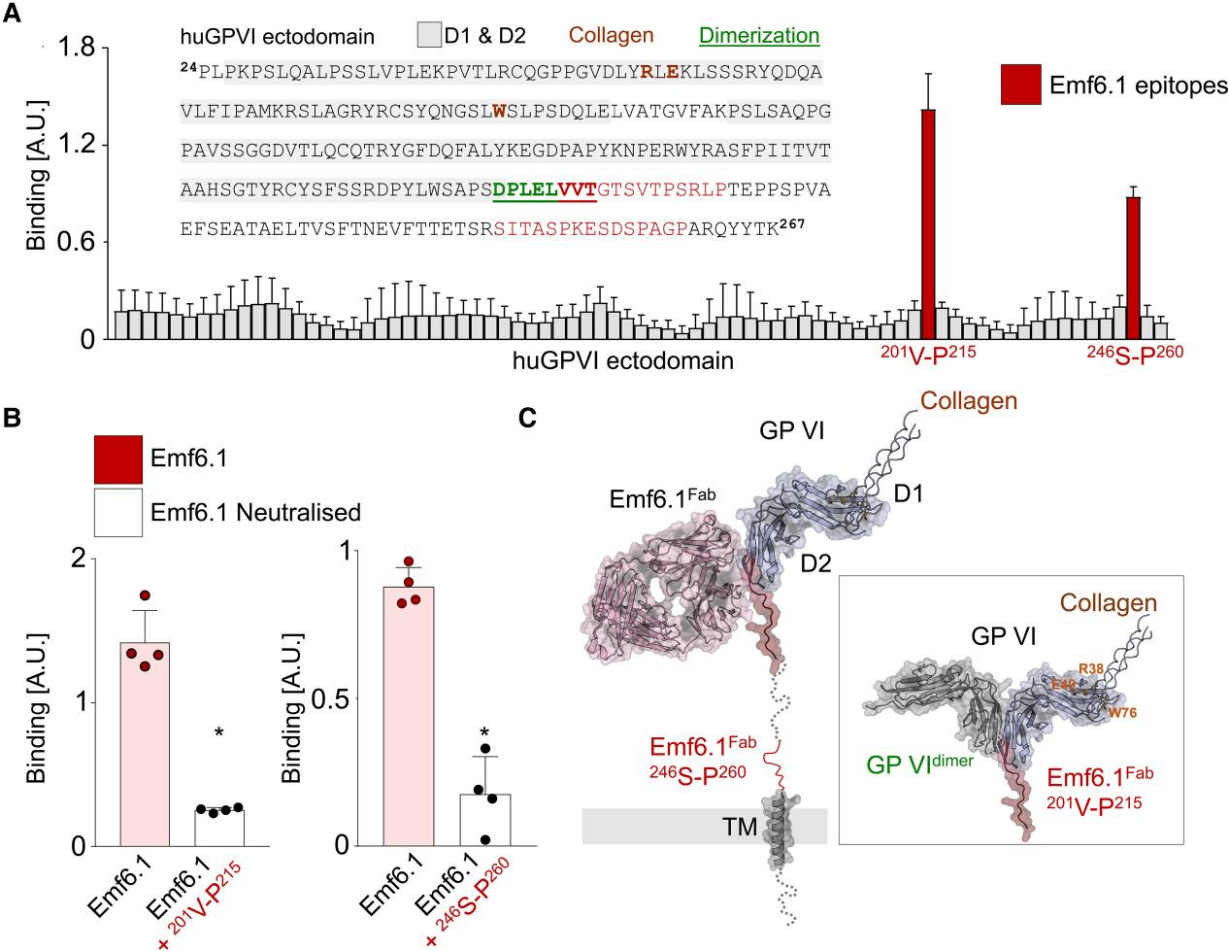
Emf6.1 binds a novel glycoprotein VI epitope. Glycoprotein VI-epitope mapping and confirmation of Emf6.1. (*A*) 15-Mer overlapping peptides covering the ectodomain (Residues 24–266) of glycoprotein VI were displayed in peptide-microarray format. Emf6.1 IgG binding was visualized by HRP-labelled anti-mouse secondary antibody. Mapping identified two distinct epitopes ^201^V-P^215^ and ^246^S-P^260^. (*B*) Emf6.1 binding is neutralized in the presence of the soluble glycoprotein VI peptide fragments ^201^V-P^215^ and ^246^S-P^260^ or vehicle solution. (*C*) Surface representation of glycoprotein VI (PDB-ID 2gi7). The Emf6.1-binding epitope (red) overlaps with the putative dimerization site (green) of glycoprotein VI but not the substrate-binding site (shown in orange for collagen PDB-ID 5ou9). The PyMOL image was generated by aligning the AF2 prediction (generated with AlphaFold) to glycoprotein VI–collagen (5ou9) and the dimer (2gi7). Data are expressed as mean ± standard deviation; significance is expressed as *P < .05

To determine the binding epitope of Emf6.1 on hGPVI, a 15-mer overlapping peptide library covering the complete hGPVI ectodomain (Residues 24–257) was synthesized and printed in microarray format. Glycoprotein VI peptide microarray binding of Emf6.1-IgG was detected by HRP-labelled anti-mouse IgG antibody and revealed a clear discontinuous binding epitope located between the D2 domain and the transmembrane helix (*Figure 5A*), more precisely on residues Val^201^ to Glu^215^ and Ser^246^ to Pro^260^ (*Figure 5A*). The critical contribution of both binding surfaces to Emf6.1 binding was validated by neutralization of Emf6.1 using the corresponding soluble peptides (*Figure 5B*). Notably, the Emf6.1 binding epitope is positioned C-terminally of all so far reported GPVI epitopes (Residues 58–187) and hence also the structurally resolved collagen-binding site (Trp^76^, Arg38, and Glu40; *Figure 5C*). This result suggests that Emf6.1 may inhibit GPVI function via a non-canonical mechanism, possibly related to dimerization of the receptor, that was structurally resolved earlier and mapped to an overlapping surface (Asp^196^-Thr^203^)^16^ (*Figure 5C*). These findings supported the hypothesis that hGPVI retains its adhesive function in the presence of Emf6.1^Fab^.

### Generation of EMA601, a fully humanized Emf6.1^Fab^ variant

To generate a non-immunogenic monovalent antibody fragment suitable for treatment of human patients, Emf6.1 murine variable domains were sequenced and canonical class and subclass complementary-determining regions (CDRs) in the VH and VL domains identified using a combination of the IMGT and Kabat numbering systems.^38,39^ The closest human germline gene V-regions were *Homo sapiens* IGHV4-4 and *Homo sapiens* IGKV1-16, respectively. Databases of human IgG and human IgK sequences were searched for comparison to the murine VH and VL domains, respectively, using BLAST search algorithms, five and four candidates, respectively, each based on a combination of framework homology, maintaining key framework residues and canonical loop structure were selected. The humanized variants were created by grafting the CDRs of the murine VH and VL into these acceptor frameworks. Each of the VH and VL domains were synthesized in-frame with human IgG1 and human IgK isotype constant domain sequences, respectively, and cloned into the mammalian transient expression plasmid pETE V2. A chimeric antibody having the murine variable domains and the human Ig constant domains (HC0:LC0), and 20 humanized variants having humanized variable domains and the human Ig constant domains (HC1-5:LC1-4) were expressed using a CHO-based transient expression system and purified by affinity chromatography. Kinetic (Octet) analysis showed that in most instances, the experimental data fit a 1:1 binding model. Under the experimental conditions used, several variants exhibited affinities higher than that of the chimeric HC0:LC0 control antibody (*K*_D_: 0.427 nM), including HC1:LC3 (*K*_D_: 0.284 nM), HC1:LC2 (*K*_D_: 0.195 nM), HC2:LC2 (*K*_D_: 0.175 nM), and HC2:LC3 (*K*_D_: 0.250 nM; *Table 1*).

**Table 1:** Kinetic parameters for antibody variants and antigen interaction

Antibody	Capture level (nm)	*k*_a_ (m^−1^s^−1^)	*k*_d_ (s^−1^)	*K*_D_ (nM)	*R* ^2^	*X* ^2^	Mean *R*_max_
*HC0 LC0*	*0*.*731*	*4.96E+05*	*2.12E−04*	*0*.*427*	*0*.*9957*	*0*.*0635*	*0*.*125*
HC1 LC1	0.709	8.04E+05	3.32E*−*04	0.413	0.9945	0.887	0.130
**HC1 LC2**	**0**.**858**	**6.28E+05**	**1.23E** *−* **04**	**0**.**195**	**0**.**9947**	**0**.**5873**	**0**.**159**
HC1 LC3	0.675	7.77E+05	2.21E*−*04	0.284	0.9977	0.0635	0.133
HC1 LC4	0.708	6.71E+05	3.33E*−*04	0.496	0.9958	0.087	0.202
HC2 LC1	0.707	7.03E+05	1.89E*−*04	0.269	0.9966	0.863	0.131
HC2 LC2	0.681	9.31E+05	1.62E*−*04	0.175	0.9969	0.0776	0.131
HC2 LC3	0.726	5.76E+05	1.44E*−*04	0.250	0.9854	0.1888	0.106
HC2 LC4	0.722	1.59E+06	1.10E*−*03	0.693	0.9775	0.2152	0.086
HC3 LC1	0.690	1.66E+06	5.87E*−*04	0.353	0.9964	0.1022	0.122
HC3 LC2	0.692	1.68E+06	3.62E*−*04	0.215	0.9943	0.1319	0.125
HC3 LC3	0.661	1.87E+06	5.61E*−*04	0.299	0.9902	0.1019	0.135
HC3 LC4	0.702	1.43E+06	6.18E*−*04	0.431	0.9942	0.1227	0.115
HC4 LC1	0.708	7.97E+05	3.39E*−*04	0.425	0.9933	0.1647	0.140
HC4 LC2	0.671	1.29E+06	5.08E*−*04	0.395	0.9962	0.0559	0.146
HC4 LC3	0.725	1.10E+06	6.55E*−*04	0.593	0.9761	0.3477	0.373
HC4 LC4	0.711	7.20E+05	4.12E*−*04	0.572	0.9947	0.0667	0.113
HC5 LC1	0.706	7.82E+05	2.61E*−*04	0.333	0.9977	0.0314	0.114
HC5 LC2	0.730	6.55E+05	2.40E*−*04	0.367	0.9905	0.112	0.224
HC5 LC3	0.704	6.86E+05	4.37E*−*04	0.637	0.9934	0.087	0.186
HC5 LC4	0.750	5.76E+05	3.19E*−*04	0.554	0.9946	0.1149	0.378

*R* ^2^ values indicate how well the fit and experimental data correlate and above 0.95 are considered a good fit; *X*^2^ is the sum of the squared deviation should be generally below 3. X^2^ is the measure of error between the experimental data and the fitted line. Italic letters: chimeric control antibody, HC0 LC0. In bold is highlighted the selected humanized variant.

**Figure 6:**
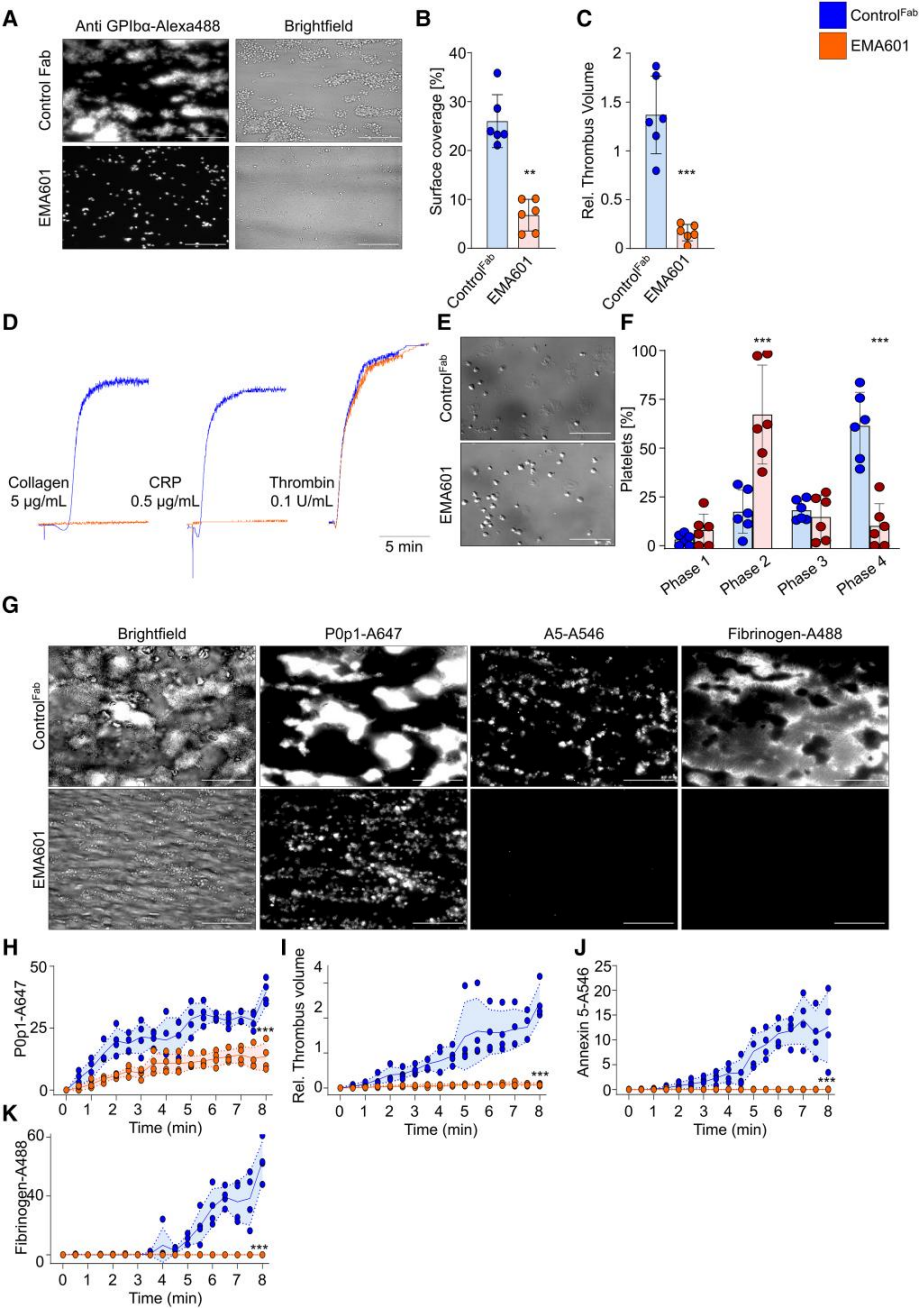
EMA601 inhibits glycoprotein VI function. (*A–C*) Assessment of platelet adhesion (*B*) and aggregate formation (*C*) on Horm collagen (50 µg/mL) under flow (1000 s^−1^) in heparinized *hGP6^tg/tg^* blood treated with 5 µg/mL EMA601 or control Fab. Values are mean ± standard deviation (*n* = 3). Unpaired, Mann–Whitney *U* test *P < .05, **P < .01. (*A*) Representative images are shown, scale 50 µM. (*D*) Aggregation responses of washed *hGP6^tg/tg^* platelets treated with either 5 µg/mL EMA601 or control Fab in light-transmission aggregometry (*n* = 3). (*E* and *F*) Washed *hGP6^tg/tg^* platelets were allowed to spread on fibrinogen (100 µg/mL) for 45 min at 37°C. DIC pictures were taken (100× objective, scale 30 µM) (*E*) and phase abundance was determined. Phase 1: adhesion; Phase 2: filopodia formation; Phase 3: lamellipodia formation; Phase 4: fully spread platelet (*F*). Values are mean ± standard deviation (*n* = 3); unpaired, Mann–Whitney *U* test *P < .05, **P < .01. (*G–K*) Assessment of platelet adhesion (*H*), thrombus formation (*I*), phosphatidylserine exposure (*J*), and fibrin/fibrinogen deposition (*K*) on Horm collagen (200 µg/mL) plus tissue factor (500 pM) under flow (1000 s^−1^) in recalcified human blood treated with 5 µg/mL EMA601 or control Fab. Values are mean ± standard deviation (*n* = 4). Unpaired, Mann–Whitney *U* test. ***P < .001. (*D*) Representative images are shown, scale 50 µm

The HC1:LC2 variant (termed EMA601) was selected for further characterization, as its GPVI-blocking effect was most potent and it consistently yielded higher amounts compared with other high-affinity binding variants. EMA601 at concentrations of 20, 10, 5, and 1 µg/mL completely inhibited aggregate formation of human platelets on collagen in the whole-blood perfusion assay (*Figure 6A–C* and Supplementary data online, *Figure S5A–G*).

Furthermore, EMA601 (1 µg/mL) abolished CRP- and collagen-induced aggregation of human platelets, whereas it had no effect on thrombin-induced responses (*Figure 6D*). Dose–response experiments further revealed that EMA601 at concentrations of 10 and 1 µg/mL potently inhibited human platelet activation/aggregation at high collagen concentrations (10 µg/mL; Supplementary data online, *Figure S5H*) and completely blocked CRP-induced aggregate formation (see Supplementary data online, *Figure S5I*). In addition, EMA601 (5 µg/mL) inhibited GPVI-dependent spreading of human platelets on fibrinogen (*Figure 6E and F*). Finally, EMA601 potently inhibited platelet deposition and thrombus formation on a collagen/TF-coated surface under flow (1000 s^−1^) and completely abolished PS exposure and fibrin/fibrinogen deposition (*Figure 6G–K*). Overall, these data demonstrated that EMA601 very potently blocks hGPVI function *in vitro*.

### EMA601 inhibits human glycoprotein VI function with >50-fold potency compared with ACT017

**Figure 7:**
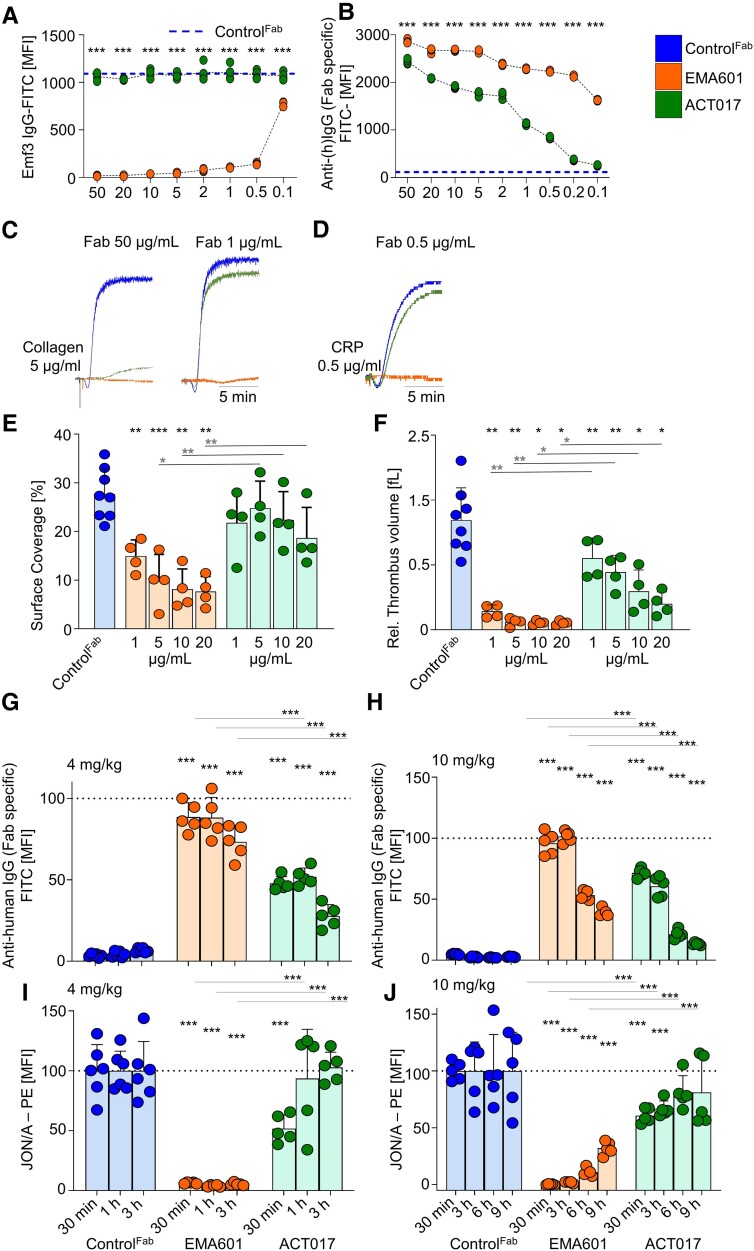
EMA601 inhibits glycoprotein VI function with >50-fold potency compared with ACT017. *hGP6^tg/tg^* diluted blood (*n* = 4) was pre-incubated with the indicated concentrations (µg/mL) of either EMA601 or ACT017 and interference with Emf3^FITC^ binding (*A*) was detected by flow cytometry. (*B*) Diluted blood of *hGP6^tg/tg^* (*n* = 4) mice was pre-incubated with the indicated concentrations of EMA601 or ACT017 and binding to platelets was detected using an anti-human IgG (Fab specific) antibody. (*C* and *D*) Aggregation traces of washed human platelets pre-treated with the indicated concentrations of EMA601 or ACT017 and stimulated with collagen (*C*) or collagen-related peptide (*D*). (*E* and *F*) Assessment of platelet adhesion (*E*) and aggregate formation (*F*) on Horm collagen (200 μg/mL) under flow (1000 s^−1^) in heparinized human blood treated with the indicated concentrations of EMA601, ACT017, or control Fab. (*G–J*) *hGP6^tg/tg^* animals were treated with 4 mg/kg (*G* and *I*) or 10 mg/kg (*H* and *J*) b.w. EMA601, ACT017, or control Fab. At the indicated time points after treatment, epitope saturation was tested using the anti-human IgG (Fab specific) antibody (*G* and *H*), while the activation of platelet αIIbβ3 integrin in response to 0.5 µg/mL collagen-related peptide was measured using JON/A-PE (*I* and *J*). Data are expressed as mean ± standard deviation; significance is expressed as *P < .05, **P < .01, ***P < .001, vs. indicated group (two-way analysis of variance followed by Bonferroni’s multiple comparison test)

To directly compare the GPVI inhibitory potencies of EMA601 and ACT017 (glenzocimab), *in vitro* and *in vivo* studies were performed. In a first set of experiments, whole blood of *hGP6^tg/tg^* mice was pre-incubated with different concentrations (ranging from 50 to 0.1 µg/mL) of EMA601 or ACT017, and epitope saturation on GPVI was tested by flow cytometry. Emf3^FITC^, which binds to an epitope in close vicinity to the Emf6.1/EMA601-binding site on hGPVI, was used to detect epitope occupancy by EMA601. While Emf3^FITC^ binding was potently inhibited by EMA601, even at a concentration of 0.5 µg/mL, ACT017 had no effect on Emf3^FITC^ binding, even at very high concentrations, confirming that EMA601 and ACT017 bind to different epitopes on hGPVI (*Figure 7A*). Next, whole blood of *hGP6^tg/tg^* mice was pre-incubated with different concentrations (ranging from 50 to 0.1 µg/mL) of EMA601 or ACT017, and the platelet-bound Fabs were detected using a fluorescently labelled anti-human IgG-Fab antibody (*n* = 4; *Figure 7B*). At all tested concentrations, EMA601 yielded significantly higher signals than ACT017, indicating a higher binding affinity of EMA601 compared with ACT017. This was further supported by the rapid decline in surface bound ACT017 at concentrations ≤2 µg/mL. Specifically, at the lowest concentrations of 0.2 and 0.1 µg/mL, ACT017 was virtually undetectable on the platelet surface, whereas EMA601 still occupied 86.2% and 57.3% of the epitopes compared with the highest tested concentration (50 µg/mL; *Figure 7B*).

Next, washed human platelets were incubated with different concentrations of EMA601, ACT017, or control Fab and their response to collagen (5 µg/mL) and CRP (0.5 µg/mL) was tested by aggregometry. EMA601 blocked collagen-induced aggregation at 1 µg/mL and still reduced it by ∼80% at 0.5 µg/mL, whereas ACT017, at concentrations of up to 10 µg/mL, had no significant inhibitory effect under these conditions (*Figure 7C and D* and Supplementary data online, *Figure S6A and B*). Of note, even at the highest tested concentration (50 µg/mL), ACT017 was unable to completely inhibit the response, demonstrating a >50-fold higher GPVI inhibitory potency of EMA601 compared with ACT017 (*Figure 7C*). Furthermore, CRP-induced aggregation was abolished at all tested concentrations of EMA601 (5, 1, and 0.5 µg/mL), whereas ACT017 showed no inhibition at 1 and 0.5 µg/mL (*Figure 7D* and Supplementary data online, *Figure S6B*).

EMA601 also inhibited aggregate formation of human platelets on collagen at arterial shear rates (1000 s^−1^) with markedly higher potency than ACT017. EMA601 significantly reduced platelet adhesion and virtually abolished aggregate formation at all tested concentrations (20, 10, 5, 1, and 0.5 µg/mL), whereas ACT017 had no significant effect on platelet adhesion under these conditions, but dose-dependently reduced thrombus volume (*Figure 7E and F* and Supplementary data online, *Figure S6C*).

Finally, to compare the GPVI inhibitory potencies of EMA601 or ACT017 *in vivo*, *hGP6^tg/tg^* mice received either 4 or 10 mg/kg b.w. of either Fab i.v. or GPVI epitope occupancy and platelet activation were monitored *ex vivo* over time. Remarkably, 4 mg/kg EMA601 led to a virtually complete receptor occupancy at 30 and 1 h, whereas 3 h after injection ∼75% of the epitopes were occupied. In contrast, at the same concentration, ACT017 occupied ∼50% of the epitopes at 30 min and 1 h, and it further decreased to ∼27% 3 h after treatment (*Figure 7G*). In agreement, CRP-induced platelet activation (JON/A^PE^ binding) was abolished in the blood of EMA601-treated mice at all time points, whereas ACT017 only significantly blocked CRP-dependent platelet activation at 30 min after injection (*Figure 7I*). At 10 mg/kg, EMA601 resulted in a complete epitope occupancy up to 3 h after injection and of ∼53% and ∼39% at 6 and 9 h, respectively. At the same dose, ACT017 showed a receptor occupancy of ∼71%, ∼61%, ∼21%, and ∼13% at 30 min, 3 h, 6 h, and 9 h, respectively (*Figure 7H*). Accordingly, 10 mg/kg EMA601 fully blocked CRP-dependent platelet activation up to 3 h, and still potently inhibited it at 6 and 9 h after injection. At the same dose, ACT017 only minimally reduced platelet activation at 1 and 3 h, whereas it had no effect at 6 and 9 h after injection (*Figure 7J*).

## Discussion

In this study, we have shown that the newly developed antibody Emf6.1^Fab^ and its humanized variant EMA601 bind to a novel membrane proximal epitope in hGPVI with very high affinity (*K*_D_: 0.427 and 0.195 nM, respectively) and very potently inhibit its function in human and *hGP6^tg/tg^* mouse platelets. Emf6.1^Fab^ treatment of *hGP6^tg/tg^* mice resulted in profound GPVI inhibition and protection from occlusive arterial thrombosis and thrombo-inflammatory cerebral infarct growth in experimental stroke, while not affecting tail bleeding times, even under conditions of complete receptor blockade (*Structured Graphical Abstract* ). These results establish EMA601 as a promising lead candidate to achieve potent, yet safe, GPVI inhibition in clinical settings.

Glycoprotein VI has emerged as a promising pharmacological target for powerful protection from arterial thrombosis and thrombo-inflammatory pathologies mainly based on initial studies in mice. The first reported function blocking anti-GPVI antibody (JAQ1) was raised against mouse GPVI.^40^*In vivo* administration of JAQ1 IgG in mice induces GPVI immunodepletion, resulting in long-term protection in a range of disease models, such as arterial thrombosis,^11^ ischaemic stroke,^6–8^ myocardial I/R injury,^13^ and LPS-induced lung injury^10^ while only moderately affecting tail bleeding time.^11^ Similar mechanisms of GPVI depletion also exist in humans,^14,41^ but persistent GPVI inhibition may not be desired in clinical settings. Based on these results, the GPVI competitor Revacept and the GPVI inhibitory Fab ACT017, which do not interfere with GPVI expression, have been developed and tested in clinical Phase 1 and Phase 2 trials. Both agents proved to be safe and well tolerated, confirming data from studies in mice and non-human primates.^19,23,42^

ACT017 (glenzocimab) and its parent mouse anti-hGPVI antibody, 9.O12, have been shown to dose-dependently reduce, but not fully block GPVI function *ex vivo*.^43^ Studies in *hGP6^tg/tg^* mice showed that 9.O12^Fab^ (4 mg/kg) significantly reduced collagen-induced aggregation *ex vivo*, but also revealed a comparably short *in vivo* half-life of the Fab of ∼2.5 h and its complete clearance within 24 h after injection.^26^ In non-human primates, 9.O12^Fab^ (4 mg/kg, i.v.) functionally blocked GPVI at *t* = 30 min after injection, and this effect was completely reversed at *t* = 24 h.^25^ In line, the first in-human study showed that administration of a single dose of 1000 mg (i.e. >16 mg/kg) ACT017 in healthy volunteers resulted in a terminal half-life of 10.2 h and an efficient reduction of collagen (2.5 µg/mL)-induced platelet aggregation for up to 8 h, albeit a complete inhibition of the receptor was not achieved at any tested dose or time point.^27^ Thus, similar to Revacept,^21,22^ ACT017 also dose dependently reduces GPVI function *in vitro*, *in vivo*, and *ex vivo*, but it does not fully block the receptor in humans^27,44^ or mice^26^ (see also *Figure 7*). In contrast, Emf6.1^Fab^/EMA601 completely blocked hGPVI-mediated activation and coagulant activity *in vitro* and *ex vivo*, thus confirming the central role of GPVI in this process.^20^ This translated into strong protection of *hGP6^tg/tg^* mice from arterial thrombosis while not affecting tail bleeding time, confirming that a complete loss of GPVI-mediated platelet activation is not associated with increased bleeding.

Besides thrombosis, GPVI also plays a central role in thrombo-inflammatory pathologies as shown in models of acute lung injury and cerebral I/R injury in ischaemic stroke where platelets mainly drive detrimental inflammation, rather than thrombus formation.^5,10,30,45,46^ In these settings, GPVI may be activated by collagen/fibrin but also other ligands, including fibronectin, vitronectin, CD147, or adiponectin (reviewed in Rayes *et al*.^14^), but further studies are needed to address their possible roles. Our study shows that Emf6.1^Fab^ treatment significantly attenuated cerebral infarct growth after tMCAO, indicating that the functional inhibition of hGPVI is protective and safe in (experimental) ischaemic stroke. In human AIS, the treatment goal by GPVI blockade would be mitigation of I/R injury (driven by thrombo-inflammation) after EVT contributing to up to 40% of the final stroke volume despite successful recanalization.^47,48^

A recently completed Phase 1b/2a trial (ACTIMIS study) assessed safety of ACT017 in patients with AIS as add-on therapy to thrombolysis/EVT. A dose of 1000 mg ACT017 was well tolerated and associated with reduced symptomatic intracranial haemorrhage and all-cause mortality compared with placebo,^23^ but the trial was not powered to assess efficacy. In line, multiple experimental studies have shown that the complete absence of GPVI (genetic ablation or immunodepletion) robustly reduces neurological damage in (hyper-)acute stroke without increasing the risk of intracranial bleeding, even when given in combination with thrombolytic treatment.^5–8^ These studies suggest that a complete blockade of GPVI function in acute thrombo-inflammatory settings will not increase the risk of bleeding but may provide more efficient protection from tissue damage compared with partial inhibition of the receptor.

Emf6.1^Fab^ efficiently blocked thrombus formation in the mechanically injured aorta as well as the FeCl_3_-injured carotid artery thrombosis. The latter model particularly mimics the situation of atherosclerotic plaque rupture, such as in symptomatic internal carotid artery (ICA) stenosis, where collagen is exposed to the blood stream and activates platelets through GPVI,^49^ leading to the formation of local thrombi and their embolization into the cerebral vasculature. Based on its potent GPVI inhibitory effect, we speculate that EMA601 has the potential to efficiently prevent brain thromboembolism during the critical time period before endarterectomy/stenting. In contrast, administration of a GPVI competitor may be less effective in this setting as shown in a recent Phase 2 trial. When Revacept was applied to patients with high-grade symptomatic ICA stenosis before intervention only a combined endpoint of neurological and cardiological events and death reached statistical significance.^50^

Notably, our data show a markedly prolonged persistence of Emf6.1^Fab^ on circulating platelets in *hGP6^tg/tg^* mice resulting in sustained inhibition of GPVI function *ex vivo* (*Figure 3D, E, and G*). One possible explanation for this extended *in vivo* activity of Emf6.1^Fab^ may be its high affinity (*K*_D_: 0.427 nM) for hGPVI which is ∼10-fold higher than the reported affinity of ACT017 (*K*_D_: 4.1 nM).^24^ Of note, the affinity of Emf6.1^Fab^ was further increased ∼2.5-fold during the humanization process with EMA601 displaying a *K*_D_ of 0.195 nM (thus ∼21-fold higher compared with ACT017). This strong inhibitory potency of EMA601 may allow for highly effective, albeit adjustable GPVI inhibition depending on the clinical setting. We expect that EMA601 achieves the desired levels of GPVI inhibition in humans at comparably lower doses compared with ACT017 and/or for longer time periods when required thereby enabling a finely tuned/tailored GPVI inhibition.

Peptide-microarray-based mapping and neutralization^51^ confirmed a novel discontinuous binding site of Emf6.1/EMA601 in hGPVI (Val^201^ to Glu^215^ and Ser^246^ to Pro^260^), between D2 and the trans-membrane helix that does not overlap with the collagen-binding interface in D1,^15^ but the GPVI dimerization residues (Val^201^-Thr^203^).^16^ We therefore speculate that Emf6.1/EMA601 may block GPVI function not primarily by interfering with ligand binding, as previously described for other GPVI antagonists,^18,52,53^ but possibly by modulating its clustering/signalling function. This would explain why Emf6.1^Fab^ potently inhibits GPVI-induced platelet activation while not fully blocking the adhesive function of the receptor (see Supplementary data online, *Figure S4F and G*).

## Conclusions

The described fully human anti-GPVI Fab, EMA601, blocks hGPVI function with very high potency and seems a promising lead candidate for the development of a safe and efficient platelet inhibitor for the treatment of acute cardio- and cerebrovascular syndromes, involving thrombus formation, such as after rupture of atherosclerotic plaques. Beyond thrombosis, GPVI drives thrombo-inflammatory complications in multiple disease settings, such as myocardial^12^ and cerebral I/R injury,^5–8,13^ acute lung injury,^10^ or glomerulonephritis^54^ and, thus, GPVI inhibition by EMA601 might impose beneficial effects on a broad range of disorders, that are not yet efficiently addressed by existing drugs.

## Supplementary Material

ehae482_Supplementary_Data

## Declarations

### Data Availability

All data are included in the manuscript as figures, tables, or supplementary material.

### Pre-registered Clinical Trial Number

None supplied.
