# Supplementary material for: The humanized platelet glycoprotein VI Fab inhibitor EMA601 protects from arterial thrombosis and ischaemic stroke in mice
Source: Eur Heart J. 2024 Aug 16;45(43):4582–97. doi: 10.1093/eurheartj/ehae482 (PMC11560278; doi:10.1093/eurheartj/ehae482)
Supplement: ehae482_Supplementary_Data [file ehae482_supplementary_data.pdf]

SUPPLEMENTAL MATERIALS

EMA601, a novel potent and safe humanized Fab inhibitor of platelet glycoprotein VI protects mice from arterial thrombosis and ischemic stroke

Short title: EMA601 - a novel potent and safe GPVI inhibitor

Stefano Navarro[1, 2], Ivan Talucci[2, 3], Vanessa Göb[1,2], Stefanie Hartmann[1], Sarah Beck[1,2], Valerie Orth[3], Guido Stoll[1], Hans M. Maric[2], David Stegner[1,2], Bernhard Nieswandt[1,2,4]

[1] Institute of Experimental Biomedicine I, Würzburg Josef-Schneider-Straße 2, 97080 Würzburg, Germany.

[2] Rudolf Virchow Center; Center for Integrative and Translational Bioimaging; University of Wuerzburg Josef-Schneider-Str. 2, 97080 Würzburg Germany.

[3] Department of Neurology, University Hospital Würzburg, Josef-Schneider-Str. 11, 97080 Würzburg, Germany

[4] EMFRET Analytics GmbH, Eibelstadt, Germany

Corresponding author:
Bernhard Nieswandt, PhD
Institute of Experimental Biomedicine, University Hospital and
Rudolf Virchow Center, University of Würzburg
Josef-Schneider-Straße 2, 97080 Würzburg, Germany
Tel.: +49 931 31 80405; Fax: +49 931 60 80405.
E-mail address: bernhard.nieswandt@virchow.uni-wuerzburg.de

MATERIALS AND METHODS

Antibodies and Reagents

Horm collagen was purchased from Takeda (Linz, Austria); the collagen-related peptide (CRP) was purchased from Cambridge Research Biochemicals (Cambridge, UK); ADP, apyrase, prostacyclin (PGI2) and fibrinogen were from Sigma Aldrich (Steinheim, Germany). Thrombin was purchased from Roche Diagnostic (Mannheim, Germany); convulxin was purchased from Enzo Life Sciences (New York, NY, USA rabbit anti-GAPDH and rat anti-mouse IgG-HRP antibodies were purchased from Sigma-Aldrich (Steinheim, Germany); U46619 was purchased from AlexisBiochemicals (Enzo Life sciences, New York, NY, USA); anti-rabbit IgG-HRP was purchased from Jackson Immuno (Suffolk, UK); goat anti-rat IgG-HRP was purchased from Dianova (Hamburg, Germany). The micro-cuvettes for aggregometry were purchased from LABITec (Ahrensburg, Germany). For the collection of human blood, S-monovettes 3.2% citrate and Safety-Fly-Needle 21G were purchased from Sarstedt (Nümbrecht, Germany). Heparin was purchased from Ratiopharm (Ulm, Germany); the 5 mL Polystyrene Round-Bottom Tubes for flow cytometry were purchased from Corning Inc. (New York, NY, USA). Emf6 was generated in parallel to the other Emf antibodies.[1] After repeated subclonings, a highly productive monoclonal subclone (Emf6.1) was isolated and further characterised (Emfret Analytics, unpublished). Glenzocimab was purchased by ProteoGenix (Schiltigheim, France). Human rt-PA (Actilyse) was purchased from Boehringer-Ingelheim (Ingelheim, Germany). Lyso-acetyl-silicate (Aspirin) was purchased from Bayer AG (Wuppertal, Germany). Iron(III) chloride hexahydrate was purchased by Carl Roth GmbH (Karlsruhe, Deutschland). The monoclonal antibodies JAQ1[2], Emf1[3], Emf2[3, 4], Emf3, Emf6.1, JON/A[5] and WUG 1.9[5] were produced, purified and derivatised in-house.

Blood Donors and Blood Collection

Blood was collected from healthy volunteers who had not been on anticoagulant or anti-platelet therapy for at least four weeks. Blood samples were obtained after written informed consent in accordance with the Declaration of Helsinki and approval by the Institutional Review Boards

of the University of Würzburg. Blood was drawn by venipuncture using butterfly needles and collected into 9 mL tubes containing 3.2% trisodium citrate. For all studies, the blood was kept at room temperature and used within four hours. All methods were performed in accordance with the relevant guidelines and regulations.

Washed human and murine platelets

Citrated human blood was collected in a 10 mL S-monovette and implemented with 2 mL of ACD pH 4.5 and then the sample was centrifuged for 20 min at 300 *g* at room temperature. Platelet-Rich-Plasma (PRP) was collected in new 15 ml tubes and supplemented with 1/10 ACD, 2 µL of apyrase/mL (0.02 U mL−1; A6410, Sigma-Aldrich) and 5 µL PGI_2/µL (0.1 µg mL−1; P6188, Sigma-Aldrich). Platelets were pelleted by centrifugation for 10 min at 500 *g*, washed twice with Tyrode's buffer (N-2-hydroxyethyl-piperazine-N02-ethanesulphonic acid; 134 mM NaCl, 0.34 mM NaH_2PO_4, 2.9 mM KCl, 12 mM $NaHCO_3$, 5 mM HEPES, 5 mM glucose, 0.35% BSA, pH 7.4) containing 2 µL apyrase/mL and 5 µL PGI_2/mL and finally resuspended at a concentration of 500,000/µL in Tyrodes buffer and kept for 30 min at 37°C before use. For the experiments, platelets were recalcified using calcified Tyrode's buffer.

Mice were anesthetised using isoflurane and bled to 1 mL in 300 µL heparin (20 U/mL in TBS, pH 7.3, Ratiopharm). The blood was centrifuged twice at 300 g for 6 min to obtain PRP. The PRP was supplemented with 0.02 U/mL apyrase (A610, Sigma-Aldrich) and 0.1 µg/mL PGI_2 (P6188, Sigma-Aldrich) and platelets were pelleted by centrifugation at 800 g for 5 min, washed twice with Tyrode's buffer (134 mM NaCl, 0.34 mM Na2HPO4, 2.9 mM KCl, 12 mM $NaHCO_3$, 5 mM HEPES, 5 mM glucose, 0.35% BSA, pH7.4) containing 0.02 U/mL apyrase and 0.1 µg/mL PGI_2. The platelets were allowed to rest for at least 30 min at 37°C prior to experiments.

Flow adhesion assay

200 µg/mL Horm collagen was coated on coverslips for 1 hour at 37°C and then blocked with 1% BSA in PBS. Heparinised human or murine blood was diluted 1:2 in Tyrode's buffer and supplemented with 2 mM Ca^{2+}. Murine platelets were labelled with an anti GPIX-Dylight 488

conjugated antibody, while human platelets were labelled using the anti-GPIbβ antibody, p0p1[6] conjugated to Dylight 488. Blood was perfused over the coverslips at a shear rate of 1,000 s^{-1} for 4 min and subsequently washed for 4 more minutes with Tyrode's buffer supplemented with calcium. After the washing step, 8 representative fields of view were imaged using a Leica DMI6000B microscope with a 63x objective (Leica Biosystems Technologies, Frankfurt, Germany). Finally, images were analysed for overall platelet surface coverage and relative thrombus volume using (fluorescent integrated density) using Fiji.17.

Coagulation flow chamber

In order to analyse the generation of thrombin under coagulating condition under flow in vitro, coverslips were coated with 50µg/mL Horm collagen for one hour at 37°C, followed by a second incubation with 500 pM tissue factor for one hour in a humid chamber. The slides were then blocked for 30 min at RT with PBS/ 1% BSA. For in situ blood recalcification, a Y-shaped silicone tube was used to pump citrated blood and recalcification buffer (32 mM $MgCl_2$ and 63 mM $CaCl_2$ in Hepes bufer pH 7.45) in the chamber. Images were taken every 30 seconds using a LEICA DMI6000B microscope with a 63x objective (Leica Biosystems Technologies, Frankfurt, Germany). Human platelets were labelled with the anti-GPIbβ antibody, p0p1-A647[6], PS-exposure was detected by binding of annexin A5-A546 (produced in-house), while fibrin/fibrinogen deposition was visualised by adding Alexa Fluor™ 488 conjugated fibrinogen from human plasma (Invitrogen, Waltham, Massachusetts, United States).

Aggregometry

Washed human or murine platelets were diluted in Tyrode's buffer supplemented with 2 mM Ca^{2+} and 100 µg/ml human fibrinogen. When thrombin was used as agonist, Tyrode's buffer was not supplemented with fibrinogen. Light-transmission aggregometry was performed using a 4-channel APACT aggregometer (LABITec Ahrensburg, Germany) under stirring conditions for 10 min after pre-incubation with the antibodies and the addition of the indicated agonists.

Spreading assay

Washed platelets were pre-incubated with the Fab fragments (with e.g. 10 µg/mL anti-GPVI Fab, or control Fab) and subsequently further diluted to 100.000/µL and then pipetted onto a 100 µg/mL fibrinogen-coated surface. The platelets were allowed to spread for 45 min at 37°C. Next, the coverslips were fixed using 4% PFA 4 for 10 min. The spread platelets were visualised using a ZEISS Axiovert (Zeiss group, Oberkochen, Germany) microscope with a 100x objective. Images were analysed using Fiji cell counter tool and phase abundance was determined by discriminating platelets based on 4 phases of spreading. Phase 1: adhesion, phase 2: filopodia formation; phase 3: lamellipodia formation; phase 4: fully spread platelet.

Measurement of platelet count and size

To assess platelet count and size, mice were bled into EDTA-coated tubes; platelet parameters were measured using an automated cell counter (ScilVet, scil animal care company GmbH, Viernheim, Germany).

Flow cytometric analysis of GPVI expression and platelet activation

For the detection of GPVI with Emf2^{FITC} and Emf3^{FITC}, murine blood diluted 1:20 in Tyrode's buffer without Ca^{2+} was pre-incubated 10 min with the indicated antibodies. For the platelet activation analysis, the murine blood was washed by centrifugation twice at 2,800 rpm for 5 min and then diluted in Tyrode's buffer with 2 mM Ca^{2+}. JON/A^{PE} (Emfret Analytics, Eibelstadt, Germany) was used to detect the activated conformation of integrin αIIbβ3 whilst P-selectin exposure was assessed by the FITC-conjugated anti-mP-selectin antibody, WUG 1.9.[5] The diluted murine blood was incubated with either CRP (10 µg/mL), thrombin (0.1 U/mL) or vehicle, together with JON/A^{PE} and anti-P-selectin^{FITC} for 12 min (6 min at 37°C and 6 min at RT). Finally, the blood was further diluted in 500 µL PBS to allow the measurement of the mean fluorescence intensity (MFI) using a FACSCelesta (BD Biosciences, Franklin Lakes, New Jersey, USA).

Mechanical injury of the abdominal aorta

To open the abdominal cavity of anesthetised mice (10 to 12-weeks old), a longitudinal midline incision was performed, and the abdominal aorta was exposed. A Doppler ultrasonic flow probe (0.5PSB699, Transonic Systems, Maastricht, The Netherlands) was placed around the vessel and thrombus formation was induced by a single firm compression (5 s) with clamps upstream of the flow probe. Blood flow was monitored over 30 min or until complete occlusion occurred (blood flow stopped for >5 min).

$FeCl_3$-induced injury of the carotid artery

To access the carotid artery of anesthetised mice (10 to 12-weeks old), a small incision of the skin directly below the jaw, down to the sternum was performed. The left carotid artery was exposed and a Doppler ultrasonic flow probe (0.5PSB699, Transonic Systems, Maastricht, The Netherlands) was placed around the vessel. The area of interest was dried to avoid any liquid interferes with the $FeCl_3$.Thrombus formation was induced by placing a 1 mm filter paper soaked in 10% $FeCl_3$ for 3 minutes on the exposed carotid artery. After the time of injury, the filter paper was carefully removed and blood flow was monitored over 30 min or until complete occlusion occurred (blood flow stopped for >5 min).[7]

Transient middle cerebral artery occlusion (tMCAO) and infarct size measurment

10- to 14-week-old, male, *hGP6$^{tg/tg}$* mice mice were injected i.v. with 4 mg/kg Emf6.1^{Fab} one hour before surgery. Mice were subjected to the tMCAO model and received a second dose of 4 mg/kg Emf6.1^{Fab} s.c. after 6 h. At 24h after tMCAO, infarct sizes were determined as previously described.[8, 9] For tMCAO, mice were anesthetised by 2% isofurane inhalation anaesthesia. A silicon rubber-coated filament (6021PK10, Doccol, Redlands, CA) was inserted in the carotid artery and advanced up to the origin of the middle cerebral artery (MCA). After 60 min, the filament was removed allowing reperfusion. 24h later, animals were sacrificed and three consecutive 2 mm thick coronal brain sections were prepared followed by staining of viable tissue with 2% 2,3,5 triphenyltetrazolium chloride (TTC; Sigma-Aldrich) for 15 min at 37

°C. Brain slices were scanned, and oedema-corrected infarct volumes were calculated by planimetry (ImageJ Software, National Institutes of Health) with the following equation: Vindirect (mm^3)= Vinfarct × (1 − (VI − VC)/VC). VI: Volume ischemic hemisphere, VC: Volume control hemisphere.

Tail bleeding assay

Mice were anesthetised by intraperitoneal injection of triple anaesthesia (Dormitor 0.5 µg/g, Midazolam 5 µg/g, and Fentanyl 0.05 µg/g body weight) and a 1-mm segment of the tail tip was removed using a scalpel. Tail bleeding was monitored by gently absorbing blood on filter paper at 20 s intervals without directly contacting the wound site. When no blood was observed on the paper, bleeding was determined to have ceased. The experiment was manually stopped after 20 min by cauterisation.

Automated µSPOT Synthesis

The GPVI ectodomain domain (residues 24-257 UniProtKB: Q9HCN6) was displayed in microarray format as 15mer overlapping peptides shifted by three residues. Peptide arrays were synthesised using MultiPep RSi robot (CEM GmbH, Kamp-Lintford, Germany) on cellulose discs containing 9-fluorenylmethyloxycarbonyl-β-alanine (Fmoc-β-Ala) linkers (average loading: 130 nmol/disc—4 mm diameter).[6] Synthesis was performed by deprotecting the Fmoc-group using 20% piperidine in dimethylformamide (DMF). Peptide chains was elongated using a coupling solution consisting of amino acids (0.5 M) with oxyma (1 M) and diisopropylmethanediimine (1 M) in DMF (1:1:1). Coupling steps were carried out for 3 times (30 min each), followed by capping (4% acetic anhydride in DMF). Cleavable peptide-amides were coupled with acid labile linker (Fmoc-rink-amide) to ensure the cleave off the cellulose support.

Cellulose discs were transferred into 96 deep-well plates for the peptides work-up. First, side chains groups were deprotected using 90% trifluoracetic acid (TFA), 2% dichloromethane, 5% H_2O and 3% triisopropylsilane (150 µL/well) for 1 h at room temperature (RT). Afterwards, the

deprotection solution was discarded and the discs were solubilised overnight (O/N) at RT, using a solvation mixture containing 88.5% TFA, 4% trifluoromethanesulfonic acid (TFMSA), 5% H_2O and 2.5% TIPS (250 µL/well). The resulting peptide-cellulose conjugates (PCCs) were precipitated in ice-cold ether (700 µL/well) and spun down at 2000× *g* for 10 min at 4°C, followed by two additional washes of the formed pellet with ice-cold ether. The resulting pellets were dissolved in DMSO (250 µL/well). PCCs solutions were mixed in 2:1 ratio with saline–sodium citrate buffer (150 mM NaCl, 15 mM trisodium citrate, pH 7.0) and transferred to a 384-well plate. For transfer of the PCC solutions to white-coated CelluSpot blank slides (76 × 26 mm, Intavis AG Peptide Services GmbH and CO. KG), a SlideSpotter (CEM GmbH) was used. After completion of the printing procedure, slides were left to dry for at least three hours.

Microarray binding assay

The microarray slides were blocked for 60 min with 5% (w/v) skimmed milk powder (Carl Roth) in phosphate-buffered saline (PBS; 137 mM NaCl, 2.7 mM KCl, 10 mM Na_2HPO_4, 1.8 mM KH_2PO_4, pH 7.4). After blocking, the slides were incubated for 30 min with Emf6.1 (5 µg/mL) in the blocking buffer, then washed 3× with PBS for 1 min. Antibody binding was detected using goat anti-mouse IgG-HRP (Thermo Fisher Cat. No. 31430, 1:5000). The chemiluminescent readout was detected with an Azure imaging system c400 (lowest sensitivity) using SuperSignal West Femto maximum sensitive substrate (Thermo Scientific GmbH, Schwerte, Germany). Epitope neutralisation was carried out by pre-incubating Emf6.1 with cleavable peptides for 30 min and then applied on the blocked slide.

Microarray binding intensities were quantified with FIJI using the “microarray profile” plugin (OptiNav Inc, Bellevue, WA, USA). After background subtraction of the mean greyscale value of the microarray surface surrounding the spots, the raw grayscale intensities for each position were obtained for the left and right sides of the internal duplicate on each microarray slide. The standard deviation (SD) between both sides was calculated.

Antibody humanisation

Emf6.1 murine variable domains were sequenced, and canonical class and sub-class complementary-determining regions (CDRs) were identified. The CDR residues in the murine VH and VL domains were identified using a combination of the IMGT and Kabat numbering systems.[10, 11] The closest human germline gene V-regions were Homo sapiens IGHV4-4 and Homo sapiens IGKV1-16, respectively. Data bases of Human IgG and Human IgK sequences were searched for comparison to the murine VH and VL domains, respectively, using BLAST search algorithms, and candidate human variable domains selected from the top 200 BLAST results each. They were reduced to four candidates each based on a combination of framework homology, maintaining key framework residues and canonical loop structure. With the CDRs of the murine VH and VL grafted into these acceptor frameworks they became the humanised variants. The humanised variants were checked to determine whether they had been humanised in accordance with WHO´s definition of humanised antibodies: The variable domains of a humanised chain has a V region amino acid sequence with, analysed as a whole is closer to human than to other species (assessed using the Immunogenetics Information System® (IMGT ®) DomainGapAlign tool).[12]

The original murine antibody VH and VL and the humanised variant sequences were screened for MHC Class II binding peptides to determine that the humanisation process had removed peptide sequences with high affinity using in silico algorithms. Each of the VH domains were synthesised in-frame with a human IgG1 isotype constant domain sequence. The Fab region was codon optimised (ATUM, USA) and the DNA sequence verified. Accordingly, each of the VL domains was synthesised in-frame with a human IgK isotype constant domain sequence. The entire light chain sequence was codon optimised (ATUM, USA) and the DNA sequence verified. DNA coding for the amino acid sequences of the variants were cloned into the mammalian transient expression plasmid pETE V2. The variants were expressed using a CHO based transient expression system and purified by affinity chromatography using AKTA chromatography equipment. Antibody purity was determined to be >95% by SDS-PAGE.

Kinetic analysis

Human GPVI (1.2 µg was immobilised on the biosensor using suitable capture surfaces and binding of Emf6.1 variants was monitored by BLI (Octet). The resulting sensograms were analysed using the supplied software (ForteBio). Time for association / dissociation: 900 / 1200; Antigen screening range (seven concentrations, dilute 3-fold): 10 – 0.014 nM. Interaction/fitting model 1:1.

Kinetic assays were performed by first capturing huGPVI protein antigen. The antigen captured biosensors were then submerged in wells containing different concentrations of Emf6.1 variants (association stage) followed by a dissociation step in running buffer. To allow for reference correction, antigen captures sensors were dipped into wells containing only buffer. This referencing provided a means of compensating for the natural dissociation of the capture antigen. Steps were performed at 25°C at a constant flow rate of 1,000 rpm. New sensors were used for each sample. Dissociation rate constants (K_D) were calculated using the ForteBio Data Analysis software. All consumables used were those recommended by ForteBio.

Supplemental Figures

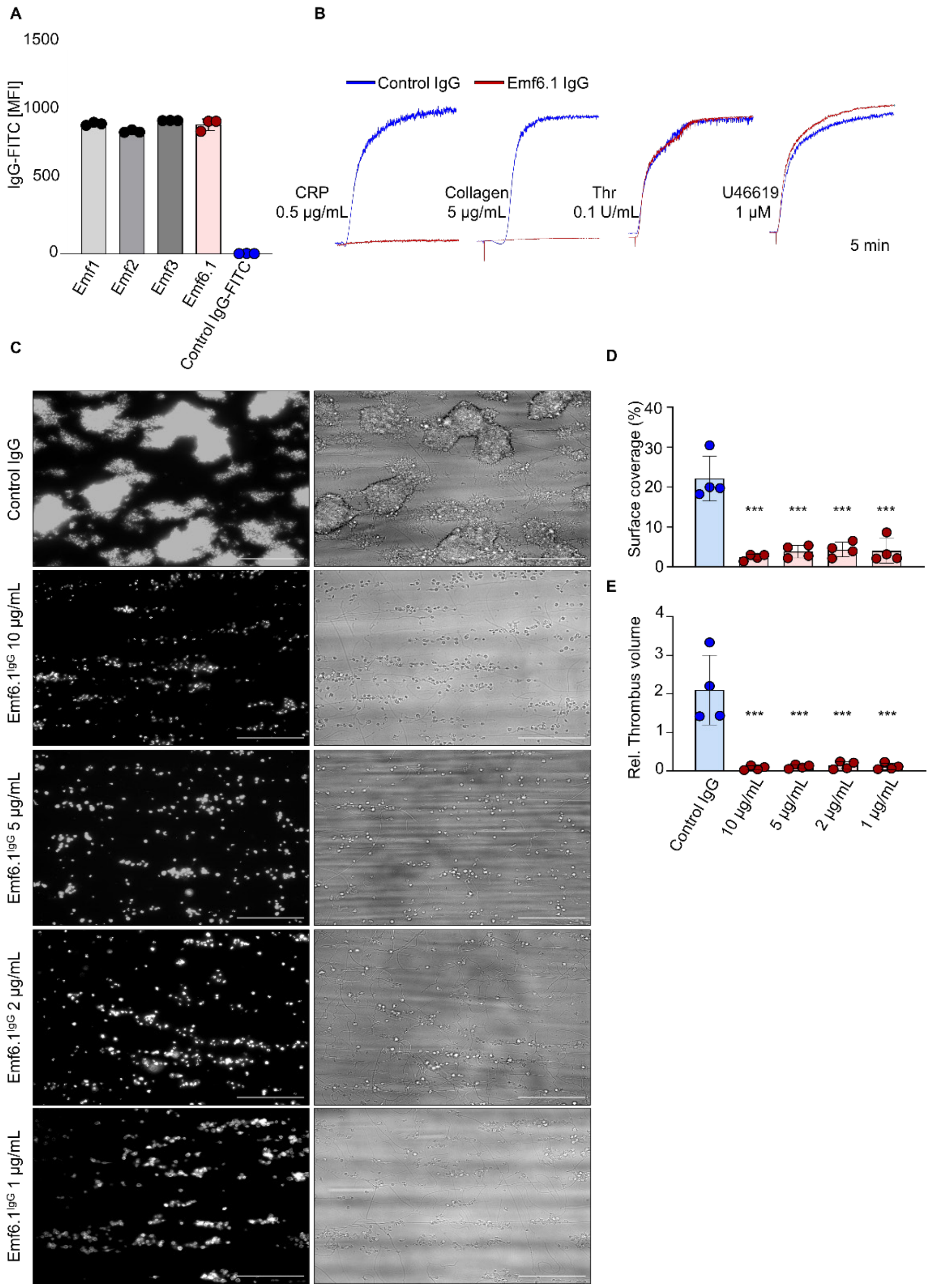

Supplemental Figure 1: Emf antibodies bind to GPVI on human platelets. (A) Flow cytometric analysis of FITC-conjugated Emf.1, Emf.2, Emf.3, Emf.6.1 or control IgG binding to GPVI on human platelets. Diluted human blood (1:10) was incubated with 10 µg/mL of the FITC conjugated respective antibodies. Values are mean ± SD (n = 3). (B) Aggregation responses of washed human platelets treated with 10 µg/mL Emf6.1 or control IgG in aggregometry assay. (C-E) Assessment of platelet adhesion (C) and relative thrombus volume formation (D) on Horm collagen (200 µg/mL) under flow (1,000 s^{-1}) in heparinised human blood treated

with 10 µg/mL Emf6.1 or control IgG. Values are mean ± SD (n = 4). ; Ordinary one-way ANOVA, *P <0.05, **P <0.01, ***P <0.001, vs. indicated group; ***P<0.001. (C) Representative images are shown, scale 50 µm.

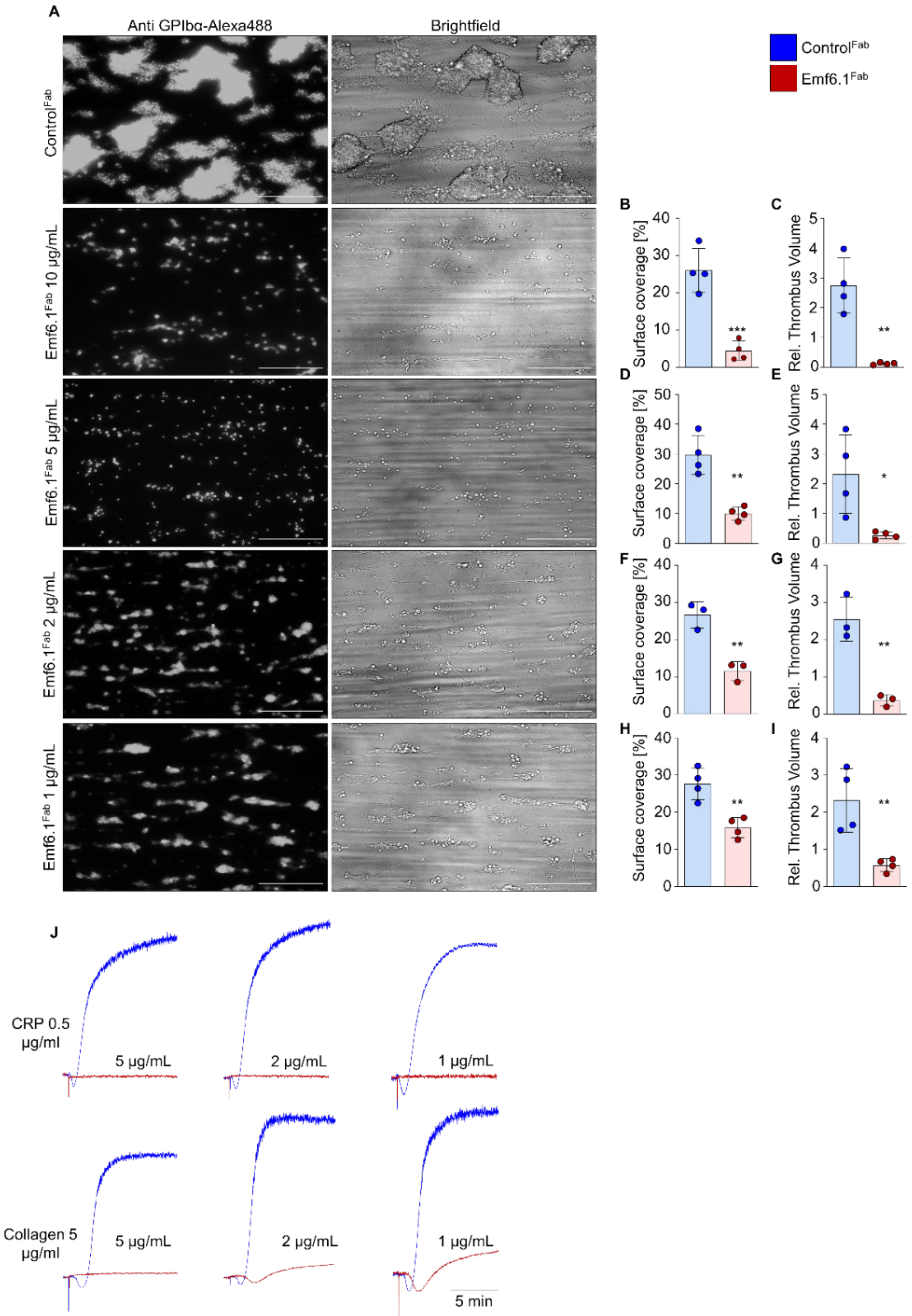

Supplemental Figure 2: Emf6.1^{Fab} inhibits GPVI-induced activation and aggregation of human platelets. (A-G) Assessment of platelet adhesion (B,D,F,H) and aggregate formation (C,E,G,I) on Horm collagen (200 µg/mL) under flow (1,000 s^{-1}) in heparinised human blood treated either with 10 µg/mL (A-C), 5 µg/mL (A, D-E), 2 µg/mL (A,F-G) or 1 µg/mL (A,H-I) Emf6.1^{Fab} or control Fab. Values are mean ± SD (n = 4). Unpaired student´s TTEST, *P <0.05, **P <0.01, ***P <0.001, vs. indicated group. (A) Representative images are shown, scale 50 µm. (J) Aggregation responses of washed human platelets in response to 0.5 µg/mL CRP or 5 µg/mL collagen treated with 5, 2 or 1 µg/mL Emf6.1^{Fab} or control Fab in light-transmission aggregometry (n = 4).

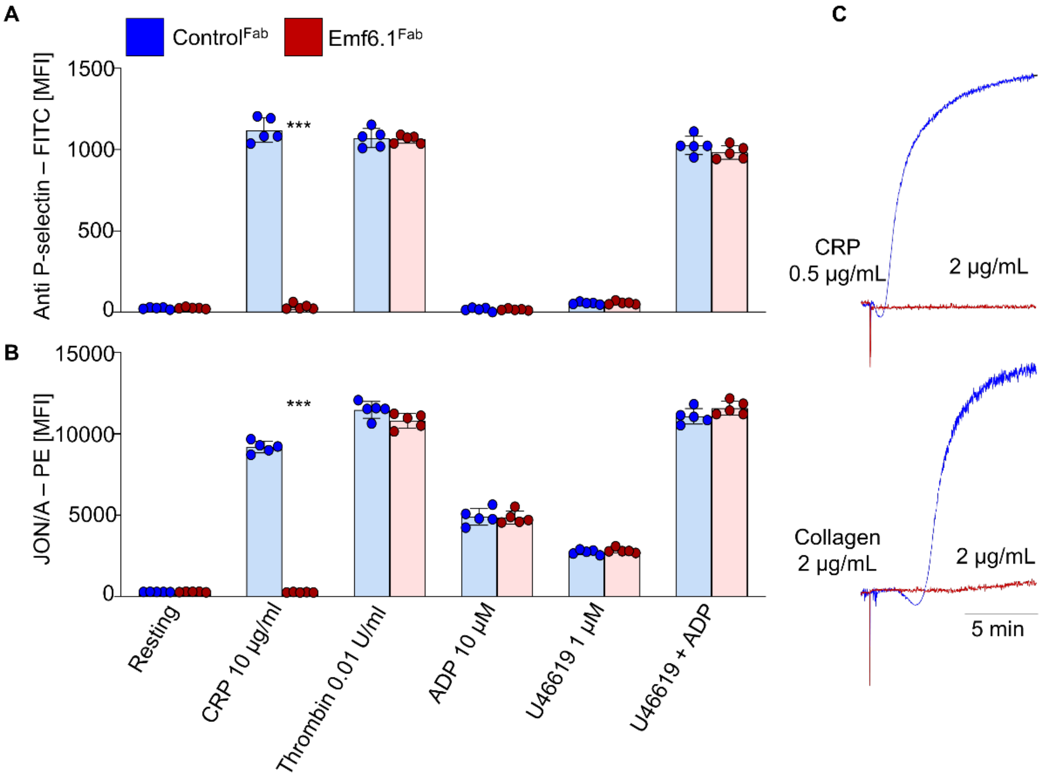

Supplemental Figure 3: Emf6.1^{Fab} inhibits GPVI-induced activation and aggregation of *hGP6$^{tg/tg}$* platelets. (A-B) Degranulation (α-P-selectin^{FITC}) (A) and activation of platelet αIIbβ3 integrin (JON/A^{PE}) (B) in *hGP6$^{tg/tg}$* platelets treated with 10 µg/ml Emf6.1^{Fab} or control^{Fab}, was determined by flow cytometry upon activation with the indicated agonists (n = 5). Two-way ANOVA followed by Bonferroni´s comparison test; ***P<0.001. (C) Aggregation responses upon CRP (0.5 µg/ml) or collagen (2 µg/mL) stimulation of washed *hGP6$^{tg/tg}$* platelets treated with either 2 µg/mL Emf.6.1^{Fab} or control^{Fab} in light-transmission aggregometry (n = 4).

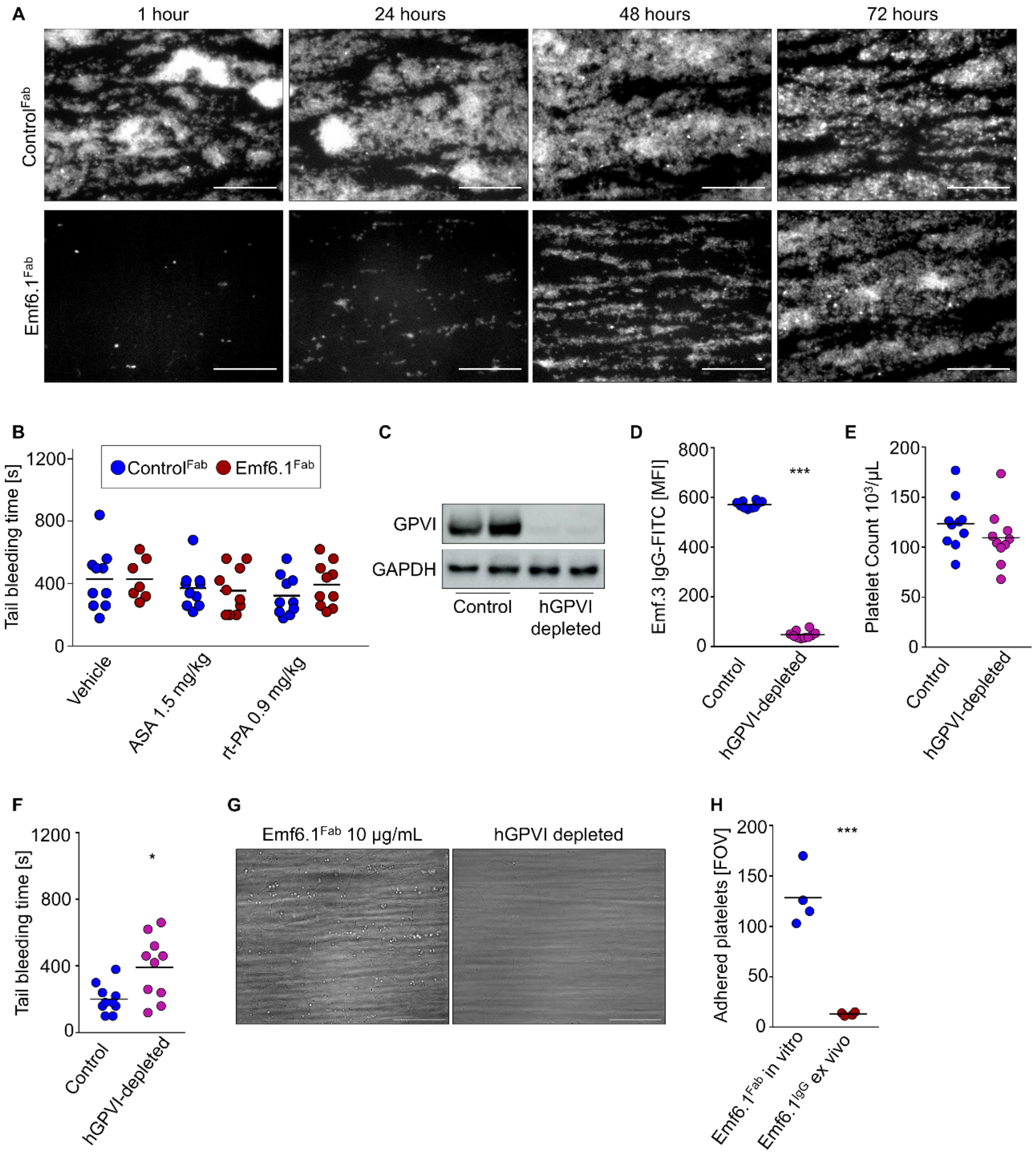

Supplemental Figure 4: Emf6.1^{Fab} treatment does not increase bleeding time and at least partly retains the adhesive function of hGPVI. (A) *hGP6$^{tg/tg}$* mice intravenously treated with 4 mg/kg b.w. Emf6.1^{Fab} or control^{Fab} and platelet adhesion and aggregate formation on Horm collagen (200 µg/mL) under flow (1,000 s^{-1}) was assessed at different time points after injection in heparinised blood.Representative images microscopic images are shown(B) *hGP6$^{tg/tg}$* mice were treated with lyso-acetylsalicylate (L-ASA, 1.5 mg/kg) or rt-PA (actilyse) in combination with either control^{Fab} or Emf6.1^{Fab}, each at4 mg/kg). Tail bleeding times were determined; each symbol represents one mouse. (C-F) *hGP6$^{tg/tg}$* mice were injected with Emf1 or control IgG and analysed on day 5 thereafter. Complete depletion of platelet hGPVI was confirmed by Western blot analysis(C) and flow-cytometry (D). Platelet count was assessed by an automated cell counter (E) and and tail bleeding times were determined (F). (G-H) Assessment of platelet

adhesion on Horm collagen (200 µg/mL) in heparinized blood; images were taken during the flow (1,000 s^{-1}) at t=2 min. Representative images (scale bar = 50 µm) (F) and quantification (G) are shown. Unpaired, Mann-U-Whitney test. *P<0.05, ***P<0.001.

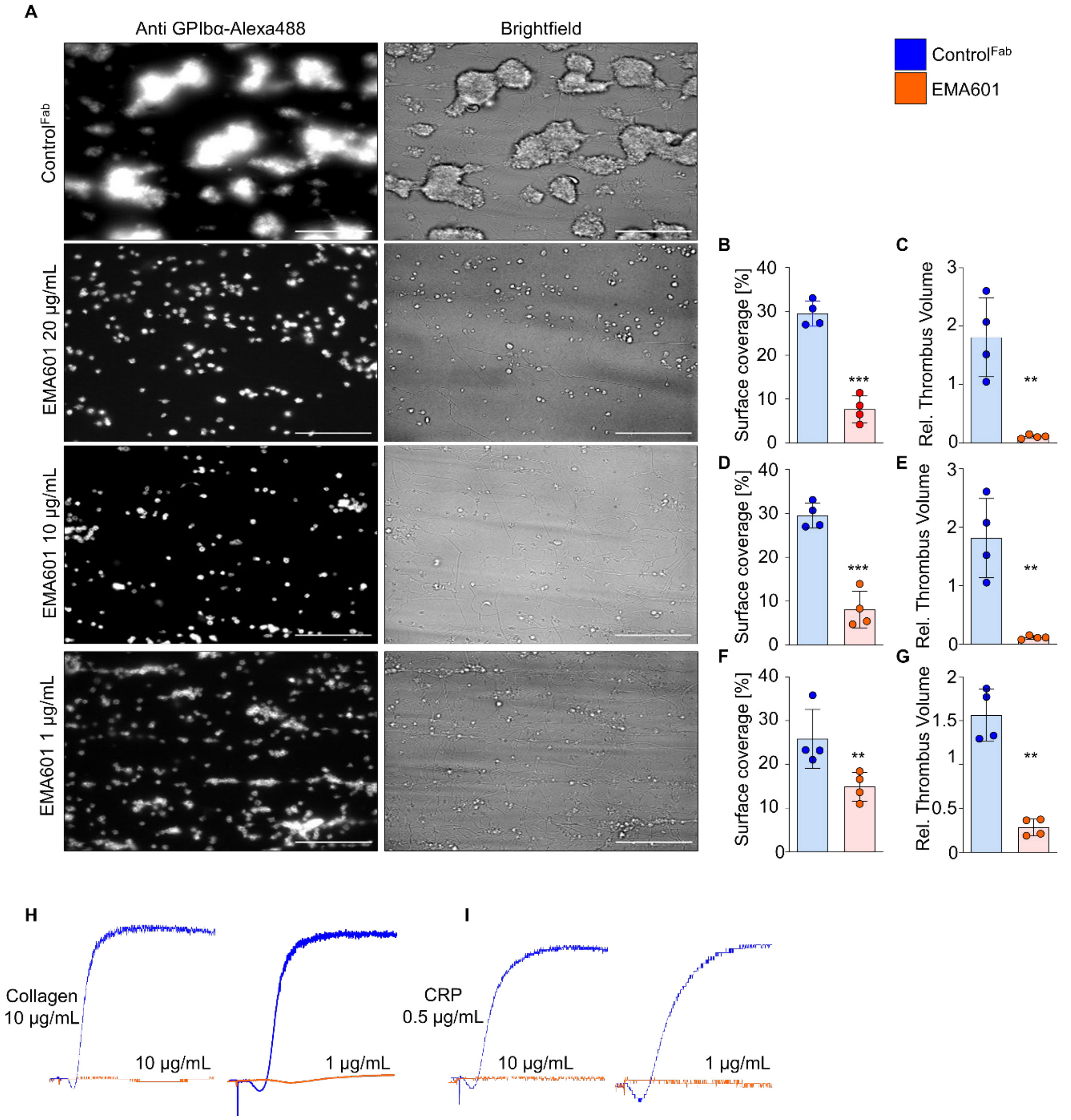

Supplemental Figure 5: EMA601 inhibits GPVI function (A-G) Assessment of platelet adhesion (B,D,F) and aggregate formation (C,E,G) on Horm collagen (200 µg/mL) under flow (1,000 s^{-1}) in heparinised human blood treated either with 20 µg/mL (A-C), 10 µg/mL (A,D-E) or 1 µg/mL (A, F-G) EMA601 or control^{Fab} fragment (n = 4). Unpaired, student´s TTest. *P<0.05, **P<0.01. (A) Representative images are shown, scale 50 µm. (H-I) Aggregation responses to 10 µg/mL collagen (H) or 0.5 µg/mL CRP (I) of washed human platelets treated

with the indicated concentrations of EMA601^{Fab} or control^{Fab} in light-transmission aggregometry (n = 4).

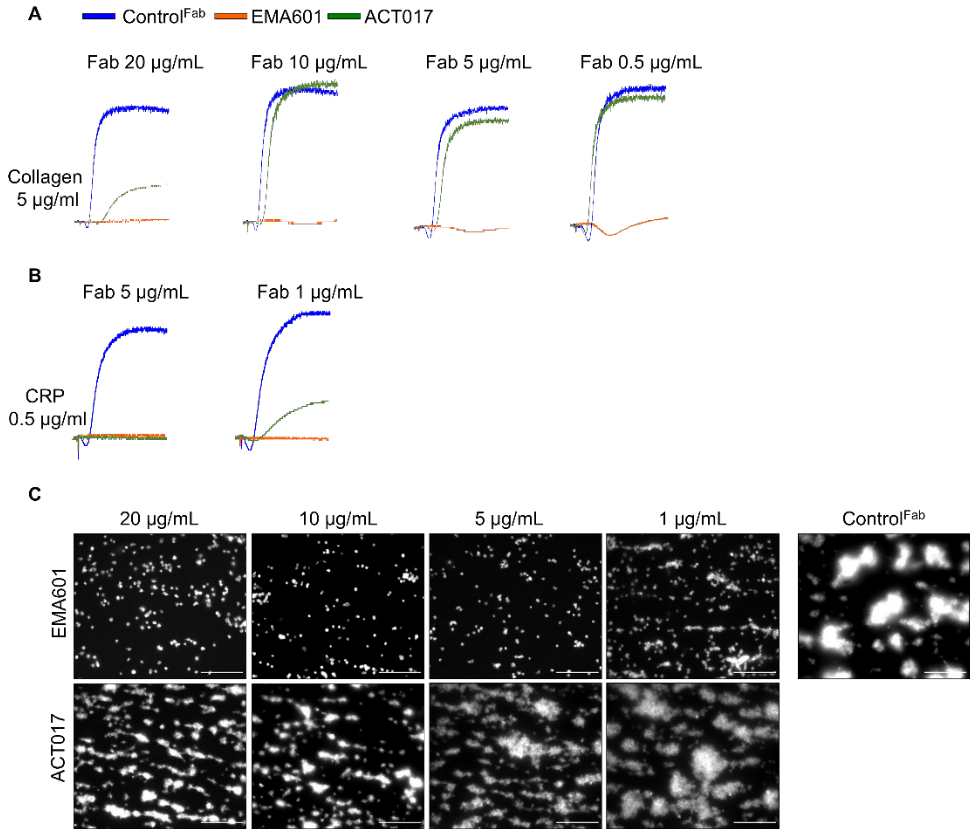

Supplemental Figure 6: EMA601 inhibits GPVI function with >50-fold potency compared to ACT017 (glenzocimab). (A-B) Representative aggregation traces and quantification showing the response of washed human platelets pre-treated with the indicated concentrations of EMA601 or ACT017 and stimulated with collagen (A) or CRP (B). (C) Representative images for the flow-adhesion assay on Horm collagen (200 µg/mL) under flow (1,000 s^{-1}) in heparinised human blood treated with the indicated concentrations of EMA601, ACT017 or control^{Fab}. Scale bar 50 µm.

Supplemental references

1. Navarro S, Stegner D, Nieswandt B, Heemskerk JWM, Kuijpers MJE. Temporal Roles of Platelet and Coagulation Pathways in Collagen- and Tissue Factor-Induced Thrombus Formation. Int J Mol Sci 2021;**23**(1).
2. Nieswandt B, Bergmeier W, Schulte V, Rackebrandt K, Gessner JE, Zirngibl H. Expression and function of the mouse collagen receptor glycoprotein VI is strictly dependent on its association with the FcRgamma chain. J Biol Chem 2000;**275**(31):23998-4002.
3. Navarro S, Starke A, Heemskerk JWM, Kuijpers MJE, Stegner D, Nieswandt B. Targeting of a Conserved Epitope in Mouse and Human GPVI Differently Affects Receptor Function. Int J Mol Sci 2022;**23**(15).
4. Navarro S, Vogtle T, Gross N, Preu J, Englert M, Nieswandt B*, et al.* Mutations of the dimerization site of glycoprotein (GP) VI result in abolished expression. Thromb Res 2023;**232**:89-92.
5. Bergmeier W, Schulte V, Brockhoff G, Bier U, Zirngibl H, Nieswandt B. Flow cytometric detection of activated mouse integrin alphaIIbbeta3 with a novel monoclonal antibody. Cytometry 2002;**48**(2):80-6.
6. Bergmeier W, Rackebrandt K, Schroder W, Zirngibl H, Nieswandt B. Structural and functional characterization of the mouse von Willebrand factor receptor GPIb-IX with novel monoclonal antibodies. Blood 2000;**95**(3):886-893.
7. Hagedorn I, Schmidbauer S, Pleines I, Kleinschnitz C, Kronthaler U, Stoll G*, et al.* Factor XIIa inhibitor recombinant human albumin Infestin-4 abolishes occlusive arterial thrombus formation without affecting bleeding. Circulation 2010;**121**(13):1510-7.
8. Gob V, Voll MG, Zimmermann L, Hemmen K, Stoll G, Nieswandt B*, et al.* Infarct growth precedes cerebral thrombosis following experimental stroke in mice. Sci Rep 2021;**11**(1):22887.
9. Schuhmann MK, Stoll G, Bieber M, Vogtle T, Hofmann S, Klaus V*, et al.* CD84 Links T Cell and Platelet Activity in Cerebral Thrombo-Inflammation in Acute Stroke. Circ Res 2020;**127**(8):1023-1035.
10. Elemento O, Lefranc MP. IMGT/PhyloGene: an on-line tool for comparative analysis of immunoglobulin and T cell receptor genes. Dev Comp Immunol 2003;**27**(9):763-79.
11. Dunbar J, Deane CM. ANARCI: antigen receptor numbering and receptor classification. Bioinformatics 2016;**32**(2):298-300.
12. Ehrenmann F, Kaas Q, Lefranc MP. IMGT/3Dstructure-DB and IMGT/DomainGapAlign: a database and a tool for immunoglobulins or antibodies, T cell receptors, MHC, IgSF and MhcSF. Nucleic Acids Res 2010;**38**(Database issue):D301-7.
